# Comparing the use of aggregate data and various methods of integrating individual patient data to network meta-analysis and its application to first-line ART

**DOI:** 10.1186/s12874-021-01254-5

**Published:** 2021-03-30

**Authors:** Steve Kanters, Mohammad Ehsanul Karim, Kristian Thorlund, Aslam H. Anis, Michael Zoratti, Nick Bansback

**Affiliations:** 1 School of Population and Public Health, University of British Columbia, 2206 E Mall, Vancouver, British Columbia Canada; 2 Centre for Health Evaluation and Outcome Science, University of British Columbia, Vancouver, Canada; 3 Departments of Health Research Methods, Evidence and Impact, McMaster University, Hamilton, Canada

**Keywords:** Individual patient data, IPD, Network meta-analyses, One-stage NMA, Two-stage NMA, Ecological fallacy, HIV, Guideline development

## Abstract

**Background:**

The 2018 World Health Organization HIV guidelines were based on the results of a network meta-analysis (NMA) of published trials. This study employed individual patient-level data (IPD) and aggregate data (AgD) and meta-regression methods to assess the evidence supporting the WHO recommendations and whether they needed any refinements.

**Methods:**

Access to IPD from three trials was granted through ClinicalStudyDataRequest.com (CSDR). Seven modelling approaches were applied and compared: 1) Unadjusted AgD network meta-analysis (NMA) – the original analysis; 2) AgD-NMA with meta-regression; 3) Two-stage IPD-AgD NMA; 4) Unadjusted one-stage IPD-AgD NMA; 5) One-stage IPD-AgD NMA with meta-regression (one-stage approach); 6) Two-stage IPD-AgD NMA with empirical-priors (empirical-priors approach); 7) Hierarchical meta-regression IPD-AgD NMA (HMR approach). The first two were the models used previously. Models were compared with respect to effect estimates, changes in the effect estimates, coefficient estimates, DIC and model fit, rankings and between-study heterogeneity.

**Results:**

IPD were available for 2160 patients, representing 6.5% of the evidence base and 3 of 24 edges. The aspect of the model affected by the choice of modeling appeared to differ across outcomes. HMR consistently generated larger intervals, often with credible intervals (CrI) containing the null value. Discontinuations due to adverse events and viral suppression at 96 weeks were the only two outcomes for which the unadjusted AgD NMA would not be selected. For the first, the selected model shifted the principal comparison of interest from an odds ratio of 0.28 (95% CrI: 10.17, 0.44) to 0.37 (95% CrI: 0.23, 0.58). Throughout all outcomes, the regression estimates differed substantially between AgD and IPD methods, with the latter being more often larger in magnitude and statistically significant.

**Conclusions:**

Overall, the use of IPD often impacted the coefficient estimates, but not sufficiently as to necessitate altering the final recommendations of the 2018 WHO Guidelines. Future work should examine the features of a network where adjustments will have an impact, such as how much IPD is required in a given size of network.

**Supplementary Information:**

The online version contains supplementary material available at 10.1186/s12874-021-01254-5.

## Background

With an ever-growing number of scientific publications, the need for meta-analysis to help make sense of the evidence continues to escalate [1]. Meta-analyses require that the included studies be sufficiently similar; otherwise resulting estimates may be biased due to imbalances between studies in the distribution of trial or patient characteristics that affect the relative effectiveness of the interventions being compared, named effect-modifiers [2]. Meta-regression has long been used to overcome such biases, as well as improve precision [3].

Meta-analyses typically consist of combining aggregate data (AgD) results from publications. As such, meta-regression most commonly consists of conducting linear regression of the study results as a function of an effect modifier, both in the aggregate. Two potential limitations to this form of meta-regression are: a limited number of data points to reliably estimate trends and risk of ecological fallacy (when trends at the trial-level do not match trends at the individual-level) [4]. A less common form of meta-regression involves using individual patient data (IPD), with or without AgD [5]. The use of IPD is less common, primarily due to the additional complications in obtaining such data [6]. Nonetheless, IPD meta-analysis can help overcome the two aforementioned limitations of AgD meta-regression [7]. Conducting meta-regression at patient-level values provide more data points, which also lends itself better to simultaneously adjusting for multiple variables [8].

Network meta-analysis (NMA) is an expansion of traditional meta-analysis that allows for the simultaneous analysis of multiple comparisons within a connected network of evidence [9]. Meta-regression is also an important technique to improve validity and precision of estimates in NMA [2, 10]. Given that NMA lends itself to larger evidence bases, the most common manner in which IPD is used in NMA is in analyses that include both IPD and AgD [11]. There are various ways by which to use IPD and AgD to conduct meta-regression, including two-stage approaches (whereby adjusted AgD are created using the IPD) [6], and one-stage approaches that integrate IPD and AgD together using hierarchical models [12–14].

In 2005, Simmonds et al. reported that 28/44 (63%) published IPD meta-analyses used the two-stage approach to IPD-AgD NMA. In a more recent 2015 review, the same researchers report roughly even use of one- and two-stage approaches, though outside of survival outcomes, the use of one-stage IPD-AgD NMA has become more popular [6]. There have also been further developments of ones-stage methods with Jackson et al. developing an expanded hierarchical method that may improve IPD-AgD meta-analysis by further reducing the risk of ecological fallacy [15, 16]. But there have not been many studies that have examined how the different types of meta-regression compare in their ability to improve analyses and conclusions. We sought to use a case study to examine if and which type (AgD or IPD) of meta-regression make such improvements.

The case study we used was a systematic literature review (SLR) and NMA that helped inform the 2016 World Health Organization (WHO) HIV clinical guidelines. The 2016 SLR found evidence of improved efficacy and tolerability of dolutegravir (DTG) relative to standard-dose efavirenz (EFV), the preferred first-line anchor treatment [17]. Following its completion, we sought IPD, independent of updating guidelines, for the comparison of AgD and IPD meta-regression methods and to see if more precise estimates might lead to stronger conclusions. In the 2016 analyses, DTG was nominally better than other treatments in its class, integrase inhibitors; however, these differences were seldomly statistically significant. In the same year, IAS-USA released its own clinical guidelines that suggested that all INSTIs were equivalent [18, 19]. We sought to further investigate this point.

The primary objective of this study, which was part of a doctoral thesis [20], was to compare the impact of using different established AgD- and IPD-based methods for meta-regression adjustments. A secondary objective was also to examine the change in outputs in the evidence synthesis of antiretroviral therapy (ART) among first-line HIV patients when including IPD – with a particular focus on the relative efficacy, safety and tolerability of DTG relative to other anchor treatments.

## Methods

### Systematic literature review

Study eligibility aligned with the review for the WHO Guideline update [21]. Briefly, eligible studies were randomized controlled trials (RCTs) comparing first-line ART regimens among adults and adolescents living with HIV. Eligible treatments were DTG, standard-dose EFV (low-dose) 400 mg efavirenz (EFV_400_), raltegravir (RAL), cobicistat-boosted elvitegravir (EVG/c), bictegravir (BIC), doravirine (DOR), rilpivirine (RPV), nevirapine (NVP), and ritonavir-boosted darunavir (DRV/r), atazanavir (ATV/r), and lopinavir (LPV/r); each in combination with a two nucleoside reverse transcriptase inhibitors (NRTI) backbone. The full PICOS (population, intervention, comparator, outcomes, study design) criteria are provided in the Additional file 1: Web-Appendix.

A comprehensive systematic search of the literature was conducted on 12 February 2018 using the following databases: MEDLINE, EMBASE, and CENTRAL (see Additional file 1: Web Appendix for search strategy*)*. Further manual searches of the 2016–2018 Conference on Retroviruses and Opportunistic Infections (CROI), the 2016 AIDS and Glasgow HIV conferences, and the 2017 International AIDS Society (IAS) conference were conducted. Additional studies were identified through a review of clinical trial registries and the reference lists of identified publications. Two investigators, working independently, scanned all titles and abstracts identified in the literature search and reviewed subsequent full-texts. A third investigator provided arbitration as needed for discrepancies. The same approach was used for data extraction.

On 15 August 2016, IPD from three RCTs available through ClinicalStudyDataRequest.com (CSDR) were formally requested. These were FLAMINGO (DRV/r + 2 NRTIs vs DTG + 2 NRTIs) [22, 23], SINGLE (DTG + ABC + XTC vs EFV + TDF + XTC) [24–27], and SPRING-2 (DTG + 2 NRTIs vs RAL + 2 NRTIs) [28, 29]. Access to the data was granted on 06 June 2017. In hindsight, there was one more eligible trial that was available at the time through this service, namely the phase 2 SPRING-1 [30, 31]; however, it was still included in the analysis through AgD.

The validity of individual RCTs was assessed using the Risk of Bias instrument, endorsed by the Cochrane Collaboration [32]. This instrument is used to evaluate 7 key domains: sequence generation; allocation concealment; blinding of participants and personnel; blinding of outcome assessors; incomplete outcome data; selective outcome reporting; and other sources of bias.

Reporting is in accordance with the preferred reporting items for systematic review and meta-analysis of individual participant data (PRISMA-IPD) guidelines [33].

### Preparation of the individual patient data

IPD were provided in a series of lengthwise tables following the Clinical Data Interchange Standards Consortium (CDISC) standards. Using these tables, an amalgamated IPD set combining all three studies was prepared. The patients were restricted to the full analysis sets, as in each of the respective trials [22, 29, 34]. The following outcomes were obtained: Viral suppression and change from baseline in CD4 cell counts at 24, 48 and 96 weeks; discontinuations, discontinuations due to adverse events, serious adverse events. There were no missing values except for CD4, for which analyses were only conducted on the observed data. Data were further verified to ensure that published results for each trial could be obtained from the IPD.

### Statistical models

Only select outcomes were used for the purpose of comparing the various statistical models of interest for conducting meta-regression adjustments with IPD and AgD. Assessing the impact on the HIV related results involved applying the preferred adjustment method to the remaining outcomes. The statistical models are presented below. Only the more complex random-effects models are presented, but both fixed- and random-effects were considered throughout.

#### AgD NMA

This served as the “baseline” results from which to draw comparisons. The model is as follows:

1 $$ {\displaystyle \begin{array}{c}{\theta}_{jk}=\left\{\begin{array}{c}{\mu}_{jb}\kern5.8em \mathrm{if}\ k=b\\ {}{\mu}_{jb}+{\delta}_{jb k}\kern3em \mathrm{if}\ k\succ b\end{array}\right.\\ {}{\delta}_{jb k}\sim Normal\left({d}_{bk},{\sigma}^2\right)=\kern0.5em Normal\left({d}_{Ak}-{d}_{Ab},{\sigma}^2\right)\\ {}{d}_{AA}=0\end{array}} $$

In this equation, *θ*_*jk*_ reflects the ‘underlying’ outcome for treatment *k* in study *j* that has been link-function-transformed to a normally distributed scale (e.g., logit link for dichotomous outcomes). *δ*_*jbk*_ is the trial-specific treatment effect of treatment *k* relative to treatment *b*. These trial-specific effects are drawn from a random-effects distribution: *δ*_*jbk*_~*N*(*d*_*bk*_, *σ*^2^). The pooled effects, *d*_*bk*_, are identified by expressing them in terms of the reference treatment A. The heterogeneity *σ*^2^ is assumed constant for all treatment comparisons.

#### AgD NMA with meta-regression

Traditional meta-regression for NMA as described in the NICE Technical Support Document 3 [2], and the statistical analysis plan (SAP) [35].

#### Two-stage IPD-AgD NMA

For these analyses, aggregate values for the DTG trials were calculated using the IPD. Specifically, mixed linear regression among the IPD was used to model each outcome adjusted for candidate covariates and provide predicted estimates of the aggregate value within the target population. The adjusted values were then simply applied to the above methods.

#### One-stage IPD-AgD NMA with and without adjustments

IPD and AgD were combined, along with meta-regression, in a single model. This has the advantage of being a single model using all data. The model is shown in eq. **(2)**, where *θ*_*ijk*_ is the link-function-transformed parameter from the likelihood function of interest for the *i*^*th*^ individual, in the *j*^*th*^ trial, treated with treatment *k*. Similarly, *η*_*jk*_ is the link-function-transformed parameter from the likelihood function for the AgD. *μ*_*jb*_ and *λ*_*jb*_ are the study-effects for the IPD and AgD, respectively. When including meta-regression adjustment, for the IPD *β*_0*j*_ is a study-specific effect of the subject-level covariate *x*_*ij*_. *β*_1*Ak*_ − *β*_1*Ab*_ reflects the interaction effects of covariate *x*_*ij*_ for treatment *k* relative to control treatment *b*. k-1 different regression coefficient *β*_1*Ak*_ will be estimated by the model. Parameters of primary interest from analyses are the pooled estimates of *d*_*Ak*_, the estimates for the heterogeneity, and treatment-by-covariate interaction effects *β*_1*Ak*_.

2 $$ {\displaystyle \begin{array}{c}\mathrm{IPD}\\ {}{\theta}_{ijk}=\left\{\begin{array}{c}{\mu}_{jb}+\sum \limits_l{\beta}_{0 lj}{x}_{lij}\kern16em \mathrm{if}\ k=b\\ {}{\mu}_{jb}+{\delta}_{jb k}+\sum \limits_l{\beta}_{0 lj}{x}_{lij}\kern0.5em +\kern0.5em \sum \limits_l\left({\beta}_{1 lAk}-{\beta}_{1 lAb}\right){x}_{lij}\kern2.75em \mathrm{if}\ k\succ b\end{array}\right.\\ {}\begin{array}{c}\mathrm{AgD}\\ {}{\eta}_{jk}=\left\{\begin{array}{c}{\lambda}_{jb}\kern16em \mathrm{if}\ k=b\\ {}{\lambda}_{jb}+{\delta}_{jb k}+\sum \limits_l\left({\beta}_{1 lAk}-{\beta}_{1 lAb}\right)x.{agg}_{lj}\kern3em \mathrm{if}\ k\succ b\end{array}\right.\\ {}\begin{array}{c}{\delta}_{jb k}\sim Normal\left({d}_{bk},{\sigma}^2\right)=\kern0.5em Normal\left({d}_{Ak}-{d}_{Ab},{\sigma}^2\right)\\ {}{d}_{AA}=0,{\beta}_{1 AA}=0\kern2em {d}_{Ak}\sim Normal\left(0,1000\right),{\beta}_{lk}={b}_l,{b}_l\sim Normal\left(0,1000\right)\end{array}\end{array}\end{array}} $$

#### Two-stage IPD-AgD NMA with empirical-priors

These models were the same as described in **(2)**, except that the regression coefficients were provided with an empirical prior that was informed by the IPD. Rather than start with the non-informative prior for *β*_1*Ak*_, the IPD were first used to estimate meta-regression coefficients using mixed-effects linear regression. The estimates and standard errors of the meta-regression were used to construct an empirical prior: ${\beta}_{1 Ak}\sim Normal\left(\hat{\beta},{prec}_{\hat{\beta}}\right)$. The idea here is to ensure that the IPD principally inform the meta-regression (potentially avoiding some ecological fallacy bias).

#### One-stage IPD-AgD NMA with hierarchical meta-regression

The final model that was considered was an expansion of one-stage IPD-AgD NMA that applies the hierarchical meta-regression adjustments first described by Jackson et al. and developed for NMA by Jansen et al. [15, 16] Unfortunately, these methods have only been developed for binomial outcomes. The model is shown in (**3)**. It shares the same notations as **(2)**.

3 $$ {\displaystyle \begin{array}{c}\mathrm{IPD}\\ {}{m}_{ij k}\sim Bernoulli\left({p}_{ij k}\right)\\ {}\begin{array}{c} logit\left({p}_{ij k}\right)=\left\{\begin{array}{c}{\mu}_{j\mathrm{b}}+{\beta}_0{x}_{ij}\kern16em \mathrm{if}\ k=b\\ {}{\mu}_{jb}+{\delta}_{jb k}+{\beta}_0{x}_{ij}+\left({\beta}_{1 Ak}-{\beta}_{1 Ab}\right){x}_{ij}\kern3em \mathrm{if}\ k\succ b\end{array}\right.\\ {}\mathrm{AgD}\\ {}\begin{array}{c}{r}_{jk}\sim Binomial\left({q}_{jk},{n}_{jk}\right)\\ {}{q}_{jk}={q}_{jk}^0\left(1-x.{agg}_j\right)+{q}_{jk}^1x.{agg}_j\\ {}\begin{array}{c} logit\left({q}_{jk}^0\right)=\left\{\begin{array}{c}{\lambda}_{jb}\kern20em \mathrm{if}\ k=b\\ {}{\lambda}_{jb}+{\delta}_{jb k}\kern16.5em \mathrm{if}\ k\succ b\end{array}\right.\\ {} logit\left({q}_{jk}^1\right)=\left\{\begin{array}{c}{\lambda}_{jb}+{\beta}_0\kern17.25em \mathrm{if}\ k=b\\ {}{\lambda}_{jb}+{\delta}_{jb k}+{\beta}_0+\left({\beta}_{1 Ak}-{\beta}_{1 Ab}\right)\kern5.5em \mathrm{if}\ k\succ b\end{array}\right.\\ {}\begin{array}{c}{\delta}_{jb k}\sim Normal\left({d}_{bk},{\sigma}^2\right)=\kern0.5em Normal\left({d}_{Ak}-{d}_{Ab},{\sigma}^2\right)\\ {}{d}_{AA}=0,{\beta}_{1 AA}=0\kern1.5em {d}_{Ak}\sim Normal\left(\mathrm{0,0.001}\right),{\beta}_{lk}={b}_l,{b}_l\sim Normal\left(0,1000\right)\end{array}\end{array}\end{array}\end{array}\end{array}} $$

The IPD part of this model is the same as that of the one-stage IPD-AgD NMA with adjustments, with the exception that *β*_0_ is not study specific but fixed across studies because it is now also used in the AgD part of the model (which reflects different studies). For the AgD part of the model, the number of events *r* in study *j* for treatment *k* is assumed to be binomially distributed with probability *q*_*jk*_ and sample size *n*_*jk*_. *q*_*jk*_ can be considered as the average probability of the response of interest for an individual in study *j* treated with intervention *k*.

The covariate adjustment values *β*_1*Ak*_ are distinct from those used in previous equations in that they are patient-level effects rather than trial-level effects. Even in the other IPD models, the effects are trial-level because they are estimated by both IPD and AgD. In **(3)** the values ${q}_{jk}^0$ and ${q}_{jk}^1$ are latent probabilities, therefore it is not possible to point identify *β*_0_ and *β*_1*Ak*_ from AgD only. As such, these are solely estimated through IPD, which removes the possibility of the ecological fallacy bias entirely.

### Statistical analyses

The following outcomes were used for the comparison of meta-regression methods: viral suppression and change from baseline in CD4 cell counts at 48 weeks (+/− 4 weeks), discontinuations, and discontinuations due to adverse events. We selected these because DTG and EFV_400_ are viewed to have as good or better efficacy and improved tolerability relative to EFV [36]. The target population was set to be the average population amongst EFV patients, the recommended preferred first-line regimen at the time. The following baseline variables were considered for covariate adjustments: CD4 cell counts, viral RNA (log-transformed), and proportion of males.

The three trials for which IPD were available tended to include healthier patients (higher baseline CD4 and lower baseline HIV RNA) and more males than the average EFV trial. In addition to being imbalanced, these factors were both plausible effect-modifiers and well-reported. Analyses consisted of comparing the modeling approaches described in the previous section. Identity link functions with Normal likelihoods were used for continuous outcomes. For dichotomous outcomes, logit link functions were use.

To assess the different models, the following measures were compared:

- Treatment-effect estimates and posterior distributions of key comparisons.
- Coefficient estimates and posterior distributions
- Deviance information criterion (DIC) value comparisons across models, as well as pD and deviance
- Between-study heterogeneity (between-study variance of the modelled outcome, e.g., log odds ratio [OR]; as calculated in the random-effects model)
- The proportion of points falling outside the lines c = 3 and c = 4 within leverage plots (the curves are of the form x^2^ + y = c). Points outside of the lines with c = 3 can generally be identified as contributing to the model’s poor fit (see TSD2) [37].
- Change in SUCRA (surface under the cumulative ranking curve) scores

The posterior distributions for treatment-effect estimates are the output that are subsequently used to draw inference and for decision-making in Bayesian modeling. Therefore, this was a primary measure of modeling impact. There were no specific hypotheses regarding how these would be affected beforehand. For comparisons in treatment-effect, the absolute effect was used because it is the most interpretable. For example, a difference of 5% in the proportion of viral suppression is more interpretable than a difference of 1.5 in the logarithm of the odds ratio. For the dichotomous variables, a difference of 1% was chosen as the threshold of minimal clinically important difference. For a change in CD4, a difference of 10 cells/mm^3^ was chosen to align with the values that were used in the WHO reviews. The SAP for this study was publicly available prior to conducting the analyses and provides further details regarding methods [35].

### Software

The parameters of the different models were estimated using a Markov Chain Monte Carlo method implemented in the JAGS software package. The first series of 30,000 iterations from the OpenBUGS sampler were discarded as ‘burn-in’, and the inferences were based on additional 50,000 iterations using two chains. For all analyses, model convergence was assessed through trace plots, density plots and Gelman-Rubin-Brooks (shrink factor) plots [38]. All analyses were performed using R version 3.4.4 (http://www.r-project.org/) and JAGS version 4.3. Code used to conduct the analyses is presented in the Additional file 1: Web Appendix.

## Results

### Evidence base

**Fig. 1:**
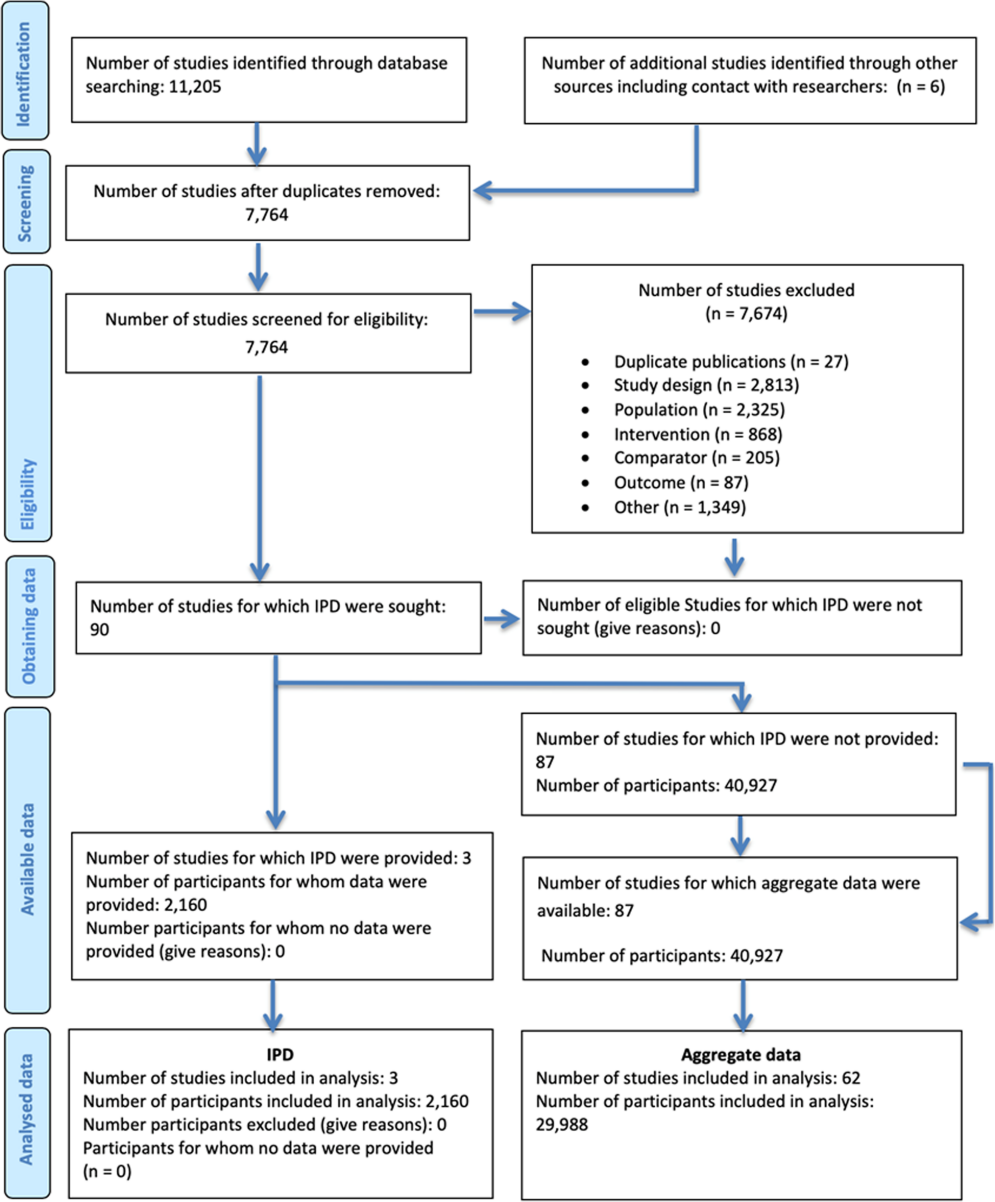
PRISMA-IPD flow diagram for identification and selection of randomized clinical trials in the evidence base

**Fig. 2:**
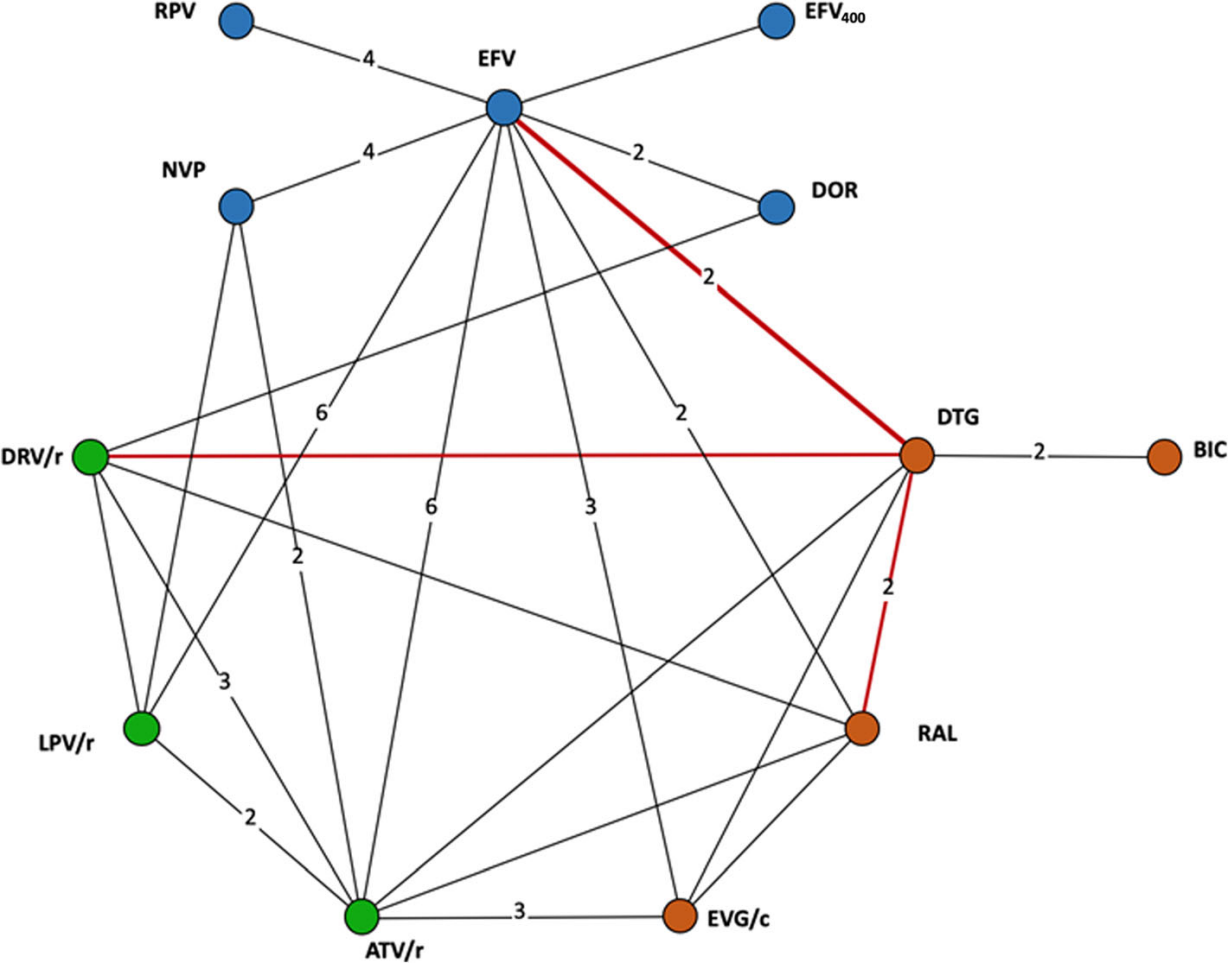
Network of evidence showing all treatments and the trial comparisons available in the evidence base. *Legend: Circles (nodes) in the diagrams represent individual treatments, lines between circles represent availability of head-to-head evidence between two treatments, and the numbers on the lines are the number of RCTs informing each head-to-head comparison. Blue: NNRTIs; Green: Protease inhibitors; Orange: Integrase inhibitors. ATV/r: ritonavir-boosted atazanavir; DRV/r: ritonavir-boosted darunavir; DTG: dolutegravir; EFV: efavirenz;* *EFV*_*400*_*: efavirenz 400; EVG/c: elvitegravir/cobicistat; LPV/r: ritonavir-boosted lopinavir; NVP: nevirapine; RAL: raltegravir; RPV: rilpivirine; BIC: bictegravir; DOR: doravirine*

Study and patient selection are presented in the PRISMA-IPD [33] flow diagram in Fig. 1. The search was conducted in three phases: the first search of AgD was conducted in May 2015 (the original SLR), a search for IPD was conducted on 15 August 2016, and then an updated search of AgD was conducted on 12 February 2018. The IPD search in 2016 involved both YODA and CDSR; however, data were only obtained through CSDR. These included 2160 patients from FLAMINGO (DRV/r vs. DTG) [22, 23], SINGLE (DTG vs EFV) [24–27], and SPRING-2 (DTG vs RAL) [28, 29]. As shown in Fig. 1, the 2160 patients for which individual patient-level data were available represent 6.5% of the total evidence base (2160/33,148), and as shown in Fig. 2, the three trials cover a total of 3 of 24 edges (12.5%; shown in red) with trials providing head-to-head evidence.

Overall study quality was generally high (i.e., low risk of bias). Exceptions were restricted to open-label trials having a high risk of bias due to blinding and some of the more recent trials that were only reported upon in posters having insufficient information to determine with certainty that the risk of bias was either low or high (Additional file 1: Web Appendix).

The patient characteristics have been described previously [21]. As shown in Figure 1–4 of the Additional file 1: Web Appendix, in addition to being the variables that were best reported in the evidence base, the covariates selected for adjustments in this study had a high degree of variability. This was especially apparent in the baseline CD4. For full posterity, the reported results by study are provided in Tables 4–5 of the Additional file 1: Web Appendix.

### Comparing meta-regression adjustments

Overall, the use of IPD appeared to have a negligible impact on the results. In each outcome, the use of IPD impacted an aspect of the results – say DIC, rankings or covariate estimates – but the aspect affected changed from one outcome to the next and tended to not be meaningful. The full set of results are shown for viral load at 48 weeks. For the remaining primary outcomes, tables and figures are presented in the Additional file 1: Web Appendix and only key highlights are focused on here.

Table 1 presents the model fit for the various models of interest for viral suppression at 48 weeks. The lowest DIC was for the unadjusted one-stage IPD-AgD NMA; however, the difference between it and the base model was not meaningful (requires a difference ≥ 3, as per SAP). The fit using the one-stage IPD-AgD NMA were considerably better than those using informative priors based on external analyses (two-stage empirical-priors approach). The use of IPD appeared to have minimal impact on the heterogeneity parameter estimate for this outcome (as calculated by the random-effects model). The proportion of observations above the third and fourth parabola in the leverage vs deviance plot tended to be stable. Nonetheless, the trend was towards having more outliers among the two-stage AgD NMA.

**Table 1:** Comparison of model selection and fit of all models considered in the analysis for viral suppression at 48 weeks as an outcome

Analyses	Model	DIC	pD	Deviance	Between study heterogeneity	prop3	prop4
AgD NMA – Unadjusted	Fixed	186.69	68.38	118.31	0.07 (0.003, 0.199)	2/116	2/116
AgD NMA meta-regression – CD4	Fixed	187.26	69.25	118.01	0.082 (0.006, 0.211)	2/116	2/116
AgD NMA meta-regression – HIV RNA	Fixed	188.79	69.38	119.41	0.07 (0.005, 0.194)	2/116	2/116
AgD NMA meta-regression – Male	Fixed	188.98	69.42	119.56	0.07 (0.003, 0.195)	2/116	2/116
Two-stage AgD NMA – CD4 + HIV RNA + Male	Fixed	189.14	68.37	120.77	0.072 (0.003, 0.204)	2/116	2/116
Two-stage AgD NMA – CD4 + Male	Fixed	188.21	68.38	119.83	0.076 (0.003, 0.208)	2/116	2/116
Two-stage AgD NMA – CD4 + HIV RNA	Fixed	193.10	68.42	124.68	0.088 (0.005, 0.228)	3/116	2/116
Two-stage AgD NMA – HIV RNA + Male	Fixed	190.15	68.44	121.71	0.078 (0.005, 0.215)	2/116	2/116
Two-stage AgD NMA – HIV RNA	Fixed	191.23	**67.67**	123.56	0.087 (0.005, 0.225)	3/116	2/116
Two-stage AgD NMA – CD4	Fixed	189.06	67.70	121.36	0.082 (0.005, 0.222)	2/116	2/116
Two-stage AgD NMA – Male	Random	205.19	82.51	122.68	0.167 (0.017, 0.306)	4/116	3/116
One-stage IPD-AgD NMA – unadjusted	Fixed	**185.11**	68.45	116.66	0.072 (0.003, 0.2)	2/116	2/116
One-stage IPD-AgD NMA – CD4 + HIV RNA + Male	Fixed	188.65	71.41	117.24	0.066 (0.003, 0.204)	2/116	2/116
One-stage IPD-AgD NMA – CD4 + Male	Fixed	188.35	70.21	118.14	0.074 (0.002, 0.206)	2/116	2/116
One-stage IPD-AgD NMA – CD4 + HIV RNA	Fixed	187.19	70.54	**116.65**	0.073 (0.004, 0.204)	2/116	2/116
One-stage IPD-AgD NMA – HIV RNA + Male	Fixed	188.90	70.58	118.32	0.074 (0.004, 0.207)	2/116	2/116
One-stage IPD-AgD NMA – CD4	Fixed	186.82	69.31	117.51	0.067 (0.001, 0.206)	2/116	2/116
One-stage IPD-AgD NMA – HIV RNA	Fixed	187.76	69.36	118.4	0.074 (0.003, 0.203)	2/116	2/116
One-stage IPD-AgD NMA – Male	Fixed	186.40	69.48	116.92	0.068 (0.004, 0.199)	2/116	2/116
Two-stage empirical-priors approach– CD4 + HIV RNA + Male	Fixed	198.56	77.47	121.09	0.104 (0.012, 0.244)	2/116	2/116
Two-stage empirical-priors – CD4 + Male	Fixed	197.69	74.18	123.51	0.108 (0.006, 0.253)	2/116	2/116
Two-stage empirical-priors – CD4 + HIV RNA	Fixed	192.22	74.75	117.47	0.071 (0.003, 0.221)	2/116	2/116
Two-stage empirical-priors – HIV RNA + Male	Fixed	195.30	74.40	120.90	0.091 (0.004, 0.23)	2/116	2/116
Two-stage empirical-priors – CD4	Fixed	191.28	71.49	119.79	0.082 (0.008, 0.215)	2/116	2/116
Two-stage empirical-priors – HIV RNA	Fixed	193.05	71.16	121.89	0.09 (0.004, 0.228)	3/116	2/116
Two-stage empirical-priors – Male	Fixed	188.59	71.81	116.78	0.075 (0.004, 0.208)	2/116	2/116
HMR IPD-AgD NMA – CD4	Fixed	185.25	70.07	115.18	0.061 (0.005, 0.181)	2/116	2/116
HMR IPD-AgD NMA – HIV RNA	Fixed	187.21	70.45	116.76	0.066 (0.001, 0.192)	2/116	2/116
HMR IPD-AgD NMA – Male	Fixed	186.34	70.44	115.90	0.072 (0.003, 0.197)	2/116	2/116

*AgD* Aggregate data, *IPD* Individual patient data, *NMA* Network meta-analysis, *DIC* Deviance information criterion, *pD* Effective number of parameters, *prop3* Proportion of observations above deviance^2^ + leverage = 3, *prop4* Proportion of observations above deviance^2^ + leverage = 4. Between-study heterogeneity obtained through the random-effects model, not the fixed-effect model if it was selected

Rankings remained generally unchanged by the model choice. Change in rankings tended to happen in the models with the highest DICs and hence those were not at risk of being favoured. Changes in the top three rankings tended to be limited to a re-ordering of the same treatments, with DTG usually remaining on top (Additional file 1: Web Appendix).

Table 2 presents the estimated effects for the comparisons of primary interest (DTG, EFV_400_ and EFV). Meta-regression adjustments based on IPD tended to lower the estimated efficacy of DTG, but almost never rendered it non-significant. The exception was the use of hierarchical meta-regression, which was limited to single variable adjustments. Importantly, these analyses included much wider credible intervals than other analyses and this was consistently observed throughout the outcomes. This aligns with results previously presented by Jansen [16]. The analyses also led to the largest shifts in estimates and these were in either direction depending on the variable of adjustment. While these methods are noted for increasing validity, we cannot conclude bias in the previous analyses on the basis of these results. Mean and maximum changes in the log-odds were large across all analyses. These changes are more easily interpretable through the change in proportions, where the maximum change was often close to 4%. The difference between 86 and 90% of patients being virally suppressed would have important implications.

**Table 2:** Comparison of comparative treatment estimates of all models considered in the analysis for viral suppression at 48 weeks as an outcome

Analyses	Model	DTG vs. EFV OR (95% CrI)	EFV_400_ vs. EFV OR (95% CrI)	DTG vs. EFV_400_ OR (95% CrI)	Mean change in log-odds	Maximum change in log-odds	Mean change in proportion	Maximum change in proportion
AgD NMA – Unadjusted	Fixed	1.85 (1.44, 2.38)	1.15 (0.75, 1.80)	1.61 (0.96, 2.68)	–	–	–	–
AgD NMA meta-regression – CD4	Fixed	1.87 (1.49, 2.39)	1.16 (0.74, 1.97)	1.62 (0.92, 2.59)	0.033	0.089	0.005	0.015
AgD NMA meta-regression – HIV RNA	Fixed	1.85 (1.45, 2.38)	1.15 (0.75, 1.80)	1.61 (0.97, 2.67)	0.001	0.004	0	0
AgD NMA meta-regression – Male	Fixed	1.85 (1.45, 2.38)	1.17 (0.73, 1.86)	1.59 (0.93, 2.73)	0.003	0.013	0	0.002
Two-stage AgD NMA – CD4 + HIV RNA + Male	Fixed	1.44 (1.12, 1.85)	1.16 (0.75, 1.80)	1.24 (0.75, 2.05)	0.063	0.266	0.01	0.042
Two-stage AgD NMA – CD4 + Male	Fixed	1.50 (1.16, 1.93)	1.15 (0.74, 1.81)	1.30 (0.78, 2.16)	0.057	0.221	0.01	0.035
Two-stage AgD NMA – CD4 + HIV RNA	Fixed	1.59 (1.23, 2.04)	1.16 (0.74, 1.80)	1.37 (0.82, 2.28)	0.056	0.183	0.009	0.029
Two-stage AgD NMA – HIV RNA + Male	Fixed	1.49 (1.16, 1.93)	1.15 (0.74, 1.79)	1.29 (0.78, 2.15)	0.065	0.238	0.009	0.036
Two-stage AgD NMA – HIV RNA	Fixed	1.34 (1.04, 1.74)	1.16 (0.74, 1.80)	1.16 (0.70, 1.94)	0.073	0.32	0.012	0.052
Two-stage AgD NMA – CD4	Fixed	1.46 (1.12, 1.91)	1.15 (0.75, 1.79)	1.27 (0.75, 2.11)	0.072	0.253	0.011	0.04
Two-stage AgD NMA – Male	Random	1.44 (1.05, 1.99)	1.15 (0.66, 2.04)	1.24 (0.65, 2.39)	0.059	0.299	0.008	0.044
One-stage IPD-AgD NMA – unadjusted	Fixed	1.60 (1.26, 2.02)	1.16 (0.75, 1.80)	1.38 (0.84, 2.27)	0.046	0.155	0.014	0.03
One-stage IPD-AgD NMA – CD4 + HIV RNA+ Male	Fixed	1.57 (1.23, 2.00)	1.10 (0.70, 1.72)	1.43 (0.86, 2.38)	0.076	0.167	0.052	0.068
One-stage IPD-AgD NMA – CD4 + Male	Fixed	1.54 (1.21, 1.95)	1.12 (0.72, 1.75)	1.37 (0.83, 2.26)	0.085	0.187	0.031	0.05
One-stage IPD-AgD NMA – CD4 + HIV RNA	Fixed	1.57 (1.24, 2.00)	1.10 (0.70, 1.72)	1.43 (0.85, 2.39)	0.076	0.166	0.03	0.048
One-stage IPD-AgD NMA – HIV RNA + Male	Fixed	1.58 (1.24, 2.01)	1.11 (0.71, 1.73)	1.41 (0.85, 2.36)	0.06	0.161	0.054	0.07
One-stage IPD-AgD NMA – CD4	Fixed	1.54 (1.22, 1.95)	1.12 (0.72, 1.75)	1.38 (0.84, 2.26)	0.083	0.181	0.02	0.037
One-stage IPD-AgD NMA – HIV RNA	Fixed	1.56 (1.23, 1.97)	1.12 (0.72, 1.75)	1.39 (0.85, 2.27)	0.066	0.174	0.022	0.039
One-stage IPD-AgD NMA – Male	Fixed	1.61 (1.27, 2.04)	1.15 (0.74, 1.80)	1.39 (0.83, 2.33)	0.042	0.142	0.008	0.026
Two-stage empirical-priors – CD4 + HIV RNA + Male	Fixed	1.60 (1.21, 2.13)	1.08 (0.68, 1.70)	1.48 (0.87, 2.54)	0.102	0.271	0.022	0.049
Two-stage empirical-priors – CD4 + Male	Fixed	1.62 (1.24, 2.12)	1.10 (0.70, 1.71)	1.48 (0.88, 2.49)	0.106	0.245	0.023	0.047
Two-stage empirical-priors – CD4 + HIV RNA	Fixed	1.65 (1.25, 2.17)	1.09 (0.70, 1.71)	1.50 (0.88, 2.54)	0.087	0.233	0.016	0.042
Two-stage empirical-priors – HIV RNA + Male	Fixed	1.58 (1.21, 2.05)	1.10 (0.70, 1.74)	1.43 (0.85, 2.40)	0.092	0.194	0.02	0.039
Two-stage empirical-priors – CD4	Fixed	1.67 (1.28, 2.17)	1.11 (0.71, 1.73)	1.50 (0.90, 2.52)	0.092	0.211	0.019	0.041
Two-stage empirical-priors – HIV RNA	Fixed	1.58 (1.24, 2.03)	1.11 (0.71, 1.74)	1.42 (0.85, 2.37)	0.083	0.178	0.021	0.04
Two-stage empirical-priors – Male	Fixed	1.58 (1.22, 2.04)	1.14 (0.73, 1.79)	1.38 (0.83, 2.32)	0.056	0.16	0.011	0.028
HMR IPD-AgD NMA – CD4	Fixed	1.34 (0.96, 1.88)	0.94 (0.57, 1.58)	1.42 (0.87, 2.31)	0.226	0.321	0.131	0.148
HMR IPD-AgD NMA – HIV RNA	Fixed	2.03 (0.92, 3.96)	1.36 (0.67, 2.57)	1.48 (0.89, 2.46)	0.16	0.221	0.120	0.170
HMR IPD-AgD NMA – Male	Fixed	2.20 (1.25, 4.02)	1.48 (0.81, 2.80)	1.48 (0.88, 2.48)	0.258	0.309	0.036	0.050

*AgD* Aggregate data, *IPD* Individual patient data, *NMA* Network meta-analysis, *EFV* Efavirenz, *DTG* Dolutegravir, *EFV*_*400*_ Low-dose efavirenz, *OR* Odds ratio, *CrI* Credible interval. Note that DTG vs EFV is estimated through a mixed treatment comparison, EFV_400_ vs EFV by direct comparison and DTG vs EFV_400_ by an indirect comparison

The estimated coefficients across the analyses are presented in Table 3. When comparing the meta-regression coefficients, the coefficient for CD4 was statistically significant in each of the IPD analyses that included it as a covariate. Moreover, its estimated effect size was consistent across the model using IPD. The coefficient estimates were notably different across AgD and IPD models, with HIV RNA leading the way.

**Table 3:** Coefficient estimates across the IPD-AgD NMA of all models considered in the analysis for viral suppression at 48 weeks as an outcome

Analyses	Model	ß_1,1_ Median (95% CrI)	ß_1,2_ Median (95% CrI)	ß_1,3_ Median (95% CrI)	ß_0,1_ Median (95% CrI)	ß_0,2_ Median (95% CrI)	ß_0,3_ Median (95% CrI)
AgD NMA meta-regression – CD4	Fixed	0.096 (−0.039, 0.216)	–	–	–	–	–
AgD NMA meta-regression – HIV RNA	Fixed	0.009 (− 0.368, 0.377)	–	–	–	–	–
AgD NMA meta-regression – Male	Fixed	0.041 (− 0.947, 1.035)	–	–	–	–	–
One-stage IPD-AgD NMA – CD4 + HIV RNA + Male	Fixed	**0.144 (0.015, 0.272)**	−0.031 (− 0.363, 0.296)	−0.237 (− 0.957, 0.429)	−0.779 (−3.481, 1.729)	0.256 (−9.87, 7.315)	−7.108 (−20.194, 17.658)
One-stage IPD-AgD NMA – CD4 + Male	Fixed	**0.135 (0.011, 0.264)**	− 0.019 (− 0.338, 0.303)	–	0.174 (− 2.918, 2.76)	−1.272 (−6.477, 3.456)	–
One-stage IPD-AgD NMA – CD4 + HIV RNA	Fixed	**0.149 (0.035, 0.269)**	−0.229 (− 0.945, 0.427)	–	−0.413 (− 2.778, 1.985)	−2.913 (− 10.851, 4.943)	–
One-stage IPD-AgD NMA – HIV RNA + Male	Fixed	−0.195 (− 0.486, 0.097)	−0.105 (− 0.791, 0.539)	–	8.999 (− 0.415, 15.519)	−19.637 (− 32.557, − 6.818)	–
One-stage IPD-AgD NMA – CD4	Fixed	**0.139 (0.028, 0.251)**	–	–	0.351 (− 1.398, 2.058)	–	–
One-stage IPD-AgD NMA – HIV RNA	Fixed	−0.182 (− 0.468, 0.104)	–	–	−0.500 (− 4.586, 3.167)	–	–
One-stage IPD-AgD NMA – Male	Fixed	− 0.014 (− 0.674, 0.607)	–	–	1.347 (− 10.007, 6.357)	–	–

For a change in baseline CD4 at 48 weeks, no models led to a meaningfully lower DIC than the unadjusted AgD NMA; however, contrary to viral suppression, here it was the two-stage models that appeared to have the best fit (DIC ranging from 182.02–184.23, relative to183.63 for the base model) among the IPD adjusted models (DIC up to 191.41 for the rest). Moreover, the two-stage analyses also reduced the number of points outside the fourth parabola in the leverage plots (0 vs. 1–3), suggesting an overall better fit to the data. The rankings were the measure most affected by choice of model for CD4. DTG was ranked first in the base case and in the IPD-AgD NMA, but EFV_400_ was ranked first when using AgD meta-regression and two-stage IPD-AgD NMA. DTG remained the favoured treatment in the one-stage and two-stage empirical-priors. With respect to the research question at hand, using a two-stage approach would impact how data were interpreted, given the change in rankings, particularly with DTG becoming a mid-ranked treatment and EFV_400_ becoming the number one ranked treatment.

Finally, with respect to CD4 most regression coefficients were not statistically significant, but similarly to the viral suppression analysis, the estimated coefficients using IPD were substantially different than those obtained through AgD meta-regression. For example, the effect of baseline HIV RNA went from 2.5 (95% CrI: − 21.2, 26.7) to 45.5 (95% CrI: 31.3, 59.9). In other words, the AgD meta-regression estimated that on average a trial initiating at a baseline HIV RNA that was one log unit higher led to a relative change in CD4 that was 2.5 cells/ml higher, whereas the one-stage IPD-AgD NMA estimated an average increase that was 45.5 cells/ml higher (keep in mind that trials did not differ by a full log unit of baseline HIV RNA).

For discontinuations, none of the models were meaningfully different from the base AgD NMA with respect to DIC. Change in estimates tended to be minimal across models. Interestingly, the exception to this was the HMR IPD-AgD NMA with adjustments for the proportion of males, which was also the model with the lowest DIC. In this model, both DTG (OR: 0.36; 95% CrI: 0.22–0.57) and EFV_400_ (OR: 0.61; 95% CrI: 0.30–1.23) were considerably more tolerable relative to EFV than in the unadjusted model, with an OR of 0.52 and 0.91, respectively.

Out of all the primary outcomes, only discontinuations due to adverse events had a model other than the unadjusted AgD NMA selected through a meaningfully lower DIC. In this case, it was the two-stage empirical priors approach with adjustments for the proportion of males that was selected with a DIC of 202.79 vs. 205.79. The one-stage analyses and two-stage empirical-priors analyses also led to a lower estimate of the between-study heterogeneity, suggesting that the adjustments helped account for between-study differences as well. The selected model shifted the principal comparison of interest from an OR of 0.28 (95% CrI: 0.17–0.44) to 0.37 (95% CrI: 0.23–0.58), but this would have little impact on decision making. With respect to absolute effects, most model adjustments led to minimal differences. This aligns well with the fact that none of the covariates were found to be statistically significant. The rankings were stable across models; however, with the selected model, DTG changed from being ranked 1st to being ranked 2nd.

### Comparative efficacy and safety

Largely, results of the analyses for the secondary outcomes led to similar impacts to those observed in the selected four outcomes above. Only in the case of viral suppression at 96 weeks, the model adjusted for baseline HIV RNA was selected (instead of the unadjusted model). As shown in Table 4, the DIC for the selected model more than 12 units smaller than the AgD NMA. The table also shows that there are other adjustments that lead to similar DICs, but in this case, we’ve selected the smallest DIC. There was no meaningful impact with respect to rankings across outcomes.

**Table 4:** Comparison of model selection and fit for viral suppression at 96 weeks

Analyses	Model	DIC	pD	Deviance	Between study	prop3	prop4
AgD NMA – Unadjusted	Fixed	111.78	**40.95**	70.83	0.114 (0.005, 0.325)	6/96	6/96
One-stage IPD-AgD NMA – CD4 + HIV RNA + Male	Fixed	102.45	43.99	58.46	0.077 (0.003, 0.254)	4/96	3/96
One-stage IPD-AgD NMA – CD4 + Male	Fixed	101.17	42.98	58.19	0.085 (0.006, 0.263)	4/96	3/96
One-stage IPD-AgD NMA – CD4 + HIV RNA	Fixed	103.91	43.03	60.88	0.094 (0.005, 0.297)	5/96	3/96
One-stage IPD-AgD NMA – HIV RNA + Male	Fixed	100.66	42.97	57.69	0.072 (0.003, 0.238)	4/96	3/96
One-stage IPD-AgD NMA – CD4	Fixed	103.22	41.91	61.31	0.101 (0.007, 0.296)	4/96	3/96
One-stage IPD-AgD NMA – HIV RNA	Fixed	**99.43**	42.04	**57.39**	0.074 (0.004, 0.241)	4/96	3/96
One-stage IPD-AgD NMA – Male	Fixed	101.22	42.02	59.2	0.088 (0.006, 0.275)	4/96	3/96

*AgD* Aggregate data, *IPD* Individual patient data, *NMA* Network meta-analysis, *DIC* Deviance information criterion, *pD* Effective number of parameters, *prop3* Proportion of observations above deviance^2^ + leverage = 3, *prop4* Proportion of observations above deviance^2^ + leverage = 4. Between-study heterogeneity obtained through the random-effects model, not the fixed-effect model if it was selected

The impact of adjustments with IPD on the actual estimates was noticeable, particularly in the case of viral suppression and change in CD4 cell counts at 96 weeks. In the case of viral suppression, the relative efficacy of DTG was reduced relative to both EFV and EFV_400_. In the selected model, the OR decreased from 1.94 (95% CrI: 1.52, 2.48) to 1.58 (95% CrI: 1.23, 2.03) relative to EFV, with a similar change relative to EFV_400_. While none of the effects changed with respect to statistical significance, the average change in modeled proportions was rather large at a mean shift of 4.1% in the selected model.

## Discussion

This study examined the change in outputs in the evidence synthesis of ART among first-line HIV patients when including IPD and compared the extent of this impact using different established IPD-based methods for meta-regression adjustments utilizing a mixture of IPD and AgD. The four methods of adjusting for covariate imbalances using IPD that were compared are: a two-stage approach, a two-stage approach with empirical priors, a one-stage approach, and hierarchical meta-regression. In this case study, none of the four methods stood out as a clearly superior approach solely on the basis of the numerical results. Nonetheless, this study does provide insights into these methods of adjustment. First, while in most analyses, the four strategies were in general agreement, there were situations where the results differed notably between the two-stage approach and other approaches, and thus the choice of method matters. Second, the hierarchical meta-regression tended to lead to the most considerable changes in effect estimates, but did so at the steep cost of reduced precision. Third, there was a remarkable difference in the coefficient estimates obtained through IPD methods and those obtained through more traditional meta-regression using AgD only, suggesting that when adjustments are needed, IPD is more appropriate to use. This study also aimed to understand the potential impact of including individual patient data for the particular application of comparing the therapeutic landscape of anchor treatments in first-line ART for the treatment of HIV. To this end, it was reassuring to find that the conclusions reached through the evidence synthesis supplemented by the individual patient data did not lead to changes that would have impacted the WHO change in guidelines that took place in December 2018 and subsequently in 2020 [39, 40].

The possibility that the limited impact of IPD on study results are due in part to the relatively small number of patients in the network providing IPD was investigated through a separate simulation study [41]. The simulation study was borne from this work. The aim of the simulation was to investigate various network factors that could be associated with the degree of benefits from including IPD, rather than to compare the various methods of adjustments, as was the goal here. The simulation study did find that the benefits of IPD are greater in small and/or sparse networks and that having too few IPD leads to negligible benefits. Another possible reason for the lack of differences between methods is a lack of ecological fallacy – whereby trends in AgD are do not reflect the trends in IPD – which is when differences between IPD and AgD adjustments are most important. Nonetheless, it is important to note that while there were minimal differences in the results between the multiple modeling methods, these do not imply that there are no differences between the methods. Several differences are still distinguishable within this case study, as further explained below.

Despite the limited impact on the interpretation of the therapeutic landscape on the basis of IPD, there are a number of advantages to the use of IPD that were observed and that have been discussed previously [6]. First, IPD more easily allows for the simultaneous adjustment of multiple covariates because it has much higher degrees of freedom. Only edges with multiple trials and differences in covariate values along those edges allow for the estimation of the covariate of interest in an AgD setting. Second, the results of this study suggested that where traditional AgD meta-regression was feasible, it was underpowered, as demonstrated by the estimated coefficients. Under the assumption that the IPD estimates based on 2160 data points are more accurate than the meta-regression adjustments based on trends among a small number of aggregate data points, the large differences seen in estimates suggest an inaccuracy among the AgD meta-regression.

There is a clear trend towards improved access to IPD and its increased use [11, 42, 43]. The most popular IPD methods have the distinct advantage of being able to adjust for unanchored networks, but require strong assumptions (no unobserved prognostic factors and effect-modifiers) and are usually limited to indirect comparisons [8, 44]. As the use of IPD increases, we can expect increased use of IPD-AgD NMA, such as the methods compared in this study. In terms of meta-analyses and network meta-analyses, there has been a shift from the predominant use of a two-stage approach to a one-stage approach [6]. As Simmonds et al. explain in their review, this is likely due to a growing familiarity with methods, improvements in computing and the recognition that regression model offers the greatest flexibility for IPD analysis [6]. The two-stage analyses in this study included the use of regression in the first stage, which was not always used in published two-stage analyses [6]. To the best of our knowledge, no study has compared the results of one-stage and two-stage IPD-AgD NMA directly. In most analyses, there were no meaningful differences in the results using either approach. Nonetheless, there were instances where one-stage and two-stage adjustments went in opposite directions. This may be a result of having the regression adjustments for the IPD done independently for each trial in the two-stage approach, rather than collectively. In the absence of differences, the two-stage approach had the advantage of being computationally less intensive and being easier to code. Conversely, the one-stage approaches had the benefit of having more easily interpretable regression coefficients and having all the analytical steps combined. Given these advantages and the fact that choice appeared to matter for some analyses, the recommendation would be to not use the traditional two-stage approach.

The choice between one-stage IPD-AgD NMA and two-stage IPD-AgD NMA with empirical-priors is less straightforward, and is ultimately dependent on the evidence base at hand. The difference between these two approaches was much more subtle. The empirical-priors method does not appear to have been used previously. As described in the methods, the motivation for its use was to isolate the coefficient estimation to the IPD (i.e., reduce the influence of the AgD on the estimation of the regression adjustments). As such, the greater difference is seen in comparisons for which there is no IPD, so that this method becomes more important when there are numerous comparisons with AgD only. Inspection of the DTG vs. EFV estimates, for which there was an IPD trial, reveals that there was general agreement between the two modeling approaches (when keeping the same covariates). On the other hand, for the EFV_400_ vs. EFV comparisons, for which there were no IPD available, the difference was notable, with the empirical-priors approach leading to a larger shift in estimates. In situations where there is an abundant number of trials and treatment comparisons that have IPD, such as in the Donegan et al. example [45], the one-stage approach, which is already well adopted, would be recommended. For networks of evidence that have few treatment comparisons with IPD trials, the empirical-priors approach is likely to maximize the IPD.

Although hierarchical meta-regression has shown some promising results, it appears that more research is still needed for these methods. Simulation work has suggested that these methods reduce bias [16], which is usually favoured over precision; however, the loss of precision observed in our work was not negligible. Moreover, it was difficult to use these methods with multiple variables at a time and the methods for use on continuous outcomes have not yet been published. Once further advancements are conducted on this method, it will be worthwhile reviewing a comparison with traditional one-stage analyses again.

As discussed above, the implications for first-line ART regimens (i.e., our secondary objective) are minimal. The evidence continues to support the DTG as the more efficient and tolerable choice of treatment. In instances where models were selected, the differences between treatments tended to be less pronounced, albeit DTG continued to perform best with respect to viral suppression, change in CD4 and tolerability.

There are several limitations to this study. First and foremost, there were very few trials for which IPD were obtained, which is a problem commonly encountered by researchers. These represented a small fraction of the trials and patients and may explain why the impact on model estimates appeared to be somewhat muted (i.e., too few IPD may get washed out in a large network). The limitation of too few data was exacerbated by the missed opportunity to get IPD for the SPRING-1 trial. The oversight was identified too far along in the process and thus could not be corrected in time. Given that this was a small Phase 2 trial that would have added a small fraction of patients to an already small sample of IPD, the impact of including or excluding its IPD is very likely to be negligible. Moreover, the SPRING-1 trial was still included in the analyses. Second, use of a single case study, particularly one with few IPD relative to the size of the network, limits the generalizability of the comparisons between the different methods of adjustments to other settings. To this end, while some conclusions have been reached, further research will be needed. Third, it is unclear whether the multiple forms of meta-regression interfered with one another. To account for differences in backbone regimens, an arm-based meta-regression was used in addition to the more traditional trial/patient-based regression adjustments, and this may have been a nuisance to the modeling process. Third, the trials for which IPD were available were principally conducted in high-income countries, which may limit the ability to make adjustments needed in studies conducted in the LMICs. Nonetheless, there tended to be a wide range of values for the covariates of interest, so this is unlikely to have been an issue [22, 23, 25]. Fourth, specific to this evidence base, there were numerous other potential effect-modifiers that were too poorly reported to allow for meta-regression adjustments to be made. These principally included ethnicity and acquisition risk groups. Finally, due to low event counts and data unavailability, not all outcomes were available for re-analysis using IPD.

## Conclusion

There are many ways in which IPD can be integrated with AgD for the purpose of NMA. Choosing the method by which to integrate these data will impact results. In most cases, the one-stage approach is recommended; however, in situations with fewer treatment comparisons that have IPD, the empirical-priors approach is a viable alternative. Further research is needed to understand whether having too few IPD can mitigate their beneficial impact. Finally, even with the revised analyses, DTG continues to demonstrate improved efficacy and tolerability over other anchor treatments.

## Supplementary Information

**Additional file 1.** Web Appendix.

## Data Availability

The datasets used and/or analysed during the current study available from the corresponding author on reasonable request (applicable only to data extracted from published manuscripts). The individual patient data that support the findings of this study are available from GlaxoSmithKline but restrictions apply to the availability of these data, which were used under license for the current study, and so are not publicly available. Data are however available from the authors upon reasonable request and with permission of GlaxoSmithKline.

## Abbreviations

AE: Adverse event
AgD: Aggregate data
AgD-NMA: Aggregate data network meta-analysis without adjustments
AgD-NMA-MR: Aggregate data network meta-analysis with meta-regression adjustments
ART: Antiretroviral therapy
ATV/r: Ritonavir-boosted atazanavir
BIC: Bictegravir
CDISC: Clinical Data Interchange Standards Consortium
CROI: Conference on Retroviruses and Opportunistic Infections
CSDR: ClinicalStudyDataRequest.com
DIC: Deviance information criterion
DOR: Doravirine
DRV/r: Ritonavir-boosted darunavir
DTG: Dolutegravir
EFV: Standard-dose efavirenz
EFV_400_: 400 mg efavirenz
EVG/c: Cobicistat-boosted elvitegravir
GENIE: Genomics Evidence Neoplasia Information Exchange
HMR: Hierarchical meta-regression
IAS: International AIDS Society
IPD: Individual patient data
IPD-NMA: Network meta-analysis with meta-regression adjustments, using both individual patient data and aggregate data
LPV/r: Ritonavir-boosted lopinavir
MAIC: Matched indirect comparisons
MCMC: Markov Chain Monte Carlo
MSE: Mean squared error
NICE: National Institute for Health Care and Excellence
NMA: Network meta-analysis
NRTI: Nucleoside reverse transcriptase inhibitors
NVP: Nevirapine
OR: Odds ratio
PAIC: Population adjusted indirect comparisons
PRISMA-IPD: Preferred reporting items for systematic review and meta-analysis of individual participant data
PSRF: Potential scale reduction factor
RAL: Raltegravir
RCT: Randomized controlled trials
RPV: Rilpivirine
SAE: Serious adverse events
SAP: Statistical analysis plan
SLR: Systematic literature review
SOAR: Supporting Open Access for Researchers
SUCRA: Surface under the cumulative ranking curve
TDF: Tenofovir disoproxil fumarate
XTC: Lamivudine or emtricitabine
YODA: Yale University Open Data Access
