# Supplementary material for: Comparing the use of aggregate data and various methods of integrating individual patient data to network meta-analysis and its application to first-line ART
Source: BMC Med Res Methodol. 2021 Mar 30;21:60. doi: 10.1186/s12874-021-01254-5 (PMC8008675; doi:10.1186/s12874-021-01254-5)
Supplement: Supplementary file 1 — Additional file 1. Web Appendix. [file 12874_2021_1254_MOESM1_ESM.docx]

| Investigation into the benefits of using IPD for the systematic literature review and network meta-analysis of first-line ART |
| --- |
| *Web Appendix* |
|  |

Table of Contents

[Additional Methods 3](#_Toc55755301)

[Motivation for selected measures to compare models 5](#_Toc55755302)

[Additional considerations 5](#_Toc55755303)

[Model code 6](#_Toc55755304)

[Additional Results 13](#_Toc55755305)

[Risk of bias 13](#_Toc55755306)

[Risk of bias across studies 15](#_Toc55755307)

[Results of individual studies 30](#_Toc55755308)

[Additional results from Stage 1 analyses 35](#_Toc55755309)

[Results from Stage 2 analyses 48](#_Toc55755310)

[References 56](#_Toc55755311)

### Additional Methods

Table 1: Scope of the literature review in PICOS form

| Criteria | Definition |
| --- | --- |
| Population | *Inclusion criteria:*   - Treatment-naïve adults and adolescents (12 years and above) living with HIV |
| Interventions | - DTG + 2NRTI - EFV_400_ + 2NRTI - Raltegravir (RAL) + 2NRTI - Elvitegravir boosted with cobicistat EVG/c + 2NRTI - Bictegravir (BIC) + 2 NRTI - Doravirine (DOR) + 2NRTI - Rilpivirine (RPV) + 2 NRTI - Nevirapine (NVP) + 2 NRTI - Darunavir boosted with ritonavir (DRV/r) + 2 NRTI - Atazanavir boosted with ritonavir (ATV/r) + 2 NRTI - Lopinavir boosted with ritonavir (LPV/r) + 2 NRTI |
| Comparator | - EFV_600_ + 2 NRTI |
| Outcomes | - Viral suppression at 48 and 96 weeks - Change from baseline CD4 at 48 and 96 weeks - Mortality - Retention - Discontinuations due to adverse events - Treatment-emergent adverse events - Severe adverse events - Development of drug resistance |
| Study design | *Inclusion criteria:*   - Randomized controlled trials (RCTs) |
| Language | Only studies published in English will be included |
| Time | Minimum follow-up time of 24 weeks |

*Note: Except for DTG, EFV_400_ and EFV_600_ treatments are required to provide indirect evidence

The search strategy presented in **Table 3** was used for MEDLINE and EMBASE via OVID. The same search strategy was adapted to the other search engines.

**Table 2: Systematic literature search strategy**

| **No.** | **Term** | **Comments** |
| --- | --- | --- |
|  | exp HIV/ or exp HIV Infection/ | HIV/AIDS terms |
|  | (HIV Infections OR HIV?1* OR HIV?2* OR HIV infect* OR human immuno?deficiency virus OR human immune?deficiency virus).ti,ab. |  |
|  | ((human immun*) AND (deficiency virus)).ti,ab. |  |
|  | (acquired immuno?deficiency syndrome OR AIDS OR acquired immunedeficiency syndrome OR acquired immune deficiency).ti,ab. |  |
|  | ((acquired immun*) AND (deficiency syndrome)).ti,ab. |  |
|  | (Salvage therapy).ti,ab. | Treatment failure and experienced |
|  | Exp Treatment Failure/ |  |
|  | (Treatment-experienced OR Antiretroviral experienced OR ART-experienced OR Experienced patients).ti,ab. |  |
|  | (treatment switch*).ti,ab. |  |
|  | (or/1-5) not (or/6-9) | **Population Final** |
|  | exp Antiretroviral Therapy, Highly Active/ | Intervention and comparators |
|  | exp Integrase Inhibitors/ |  |
|  | exp HIV Reverse Transcriptase/ |  |
|  | exp Reverse Transcriptase Inhibitors/ |  |
|  | Exp Anti-HIV Agents/ |  |
|  | exp HIV Protease Inhibitors/ |  |
|  | (atazanavir OR Reyataz OR a603019 OR BMS-232632 or atv*).ti,ab. |  |
|  | (cobicistat OR GS-9350 OR Tybost).ti,ab. |  |
|  | (dolutegravir OR Tivicay OR a613043 OR S?GSK1349572 OR GSK1349572).ti,ab. |  |
|  | (darunavir OR Prezista OR TMC114 OR a607042 or drv*).ti,ab. |  |
|  | (Elvitegravir OR GS-9137 OR Vitekta).ti,ab. |  |
|  | (emtricitabine OR Emtriva OR Coviracil OR a604004).ti,ab. |  |
|  | (lopinavir OR ABT-378 OR a602015 or lpv*).ti,ab. |  |
|  | (nevirapine OR Viramune OR a600035).ti,ab. |  |
|  | (ritonavir OR Norvir OR a696029).ti,ab. |  |
|  | (raltegravir OR Isentress OR MK-0518 OR a608004).ti,ab. |  |
|  | (efavirenz OR Efavir OR Sustiva OR Stocrin OR Efcure OR Efferven OR Estiva OR Evirenz OR Viranz OR a699004).ti,ab. |  |
|  | (Trizivir OR Aluvia OR Kaletra OR Stribild OR triumeq).ti,ab. |  |
|  | or/11-28 | **Intervention and comparators final** |
|  | (Randomized Controlled Trial or Controlled Clinical Trial).pt. | Randomized controlled trial terms |
|  | (Clinical Trial or Clinical Trial, Phase II or Clinical Trial, Phase III or Clinical Trial, Phase IV).pt. |  |
|  | Multicenter Study.pt. |  |
|  | Randomized Controlled Trial/ or Randomized Controlled Trials as Topic/ or "Randomized Controlled Trial (topic)"/ |  |
|  | Controlled Clinical Trial/ or Controlled Clinical Trials as Topic/ or "Controlled Clinical Trial (topic)"/ |  |
|  | Clinical Trial/ or Phase 2 Clinical Trial/ or Phase 3 Clinical Trial/ or Phase 4 Clinical Trial/ |  |
|  | Clinical Trials as Topic/ or Clinical Trials, Phase II as Topic/ or Clinical Trials, Phase III as Topic/ or Clinical Trials, Phase IV as Topic/ |  |
|  | "Clinical Trial (topic)"/ or "Phase 2 Clinical Trial (topic)"/ or "Phase 3 Clinical Trial (topic)"/ or "Phase 4 Clinical Trial (topic)"/ |  |
|  | or/30-37 | **Study design final** |
|  | 10 and 29 and 38 | **Complete Search** |
|  | (healthy adj3 volunteer*).ti,ab. | Features of undesired publications |
|  | (healthy adj3 subject*).ti,ab. |  |
|  | (cohort or observational study or case-control*).ti,ab. |  |
|  | 39 not (40 or 41 or 42) |  |
|  | 43 not (cost minimi* or cost-utilit* or health utility* or economic evaluation* or economic review* or cost outcome or cost analys?s or economic analys?s or budget* impact analys?s).ti,ab. |  |
|  | 44 not (review or letter or meta-analysis or case report or case series or posters or News or Newspaper article or meeting abstracts or lectures or interview or historical article or handbooks or guidelines or guidebooks or essays or editorial or comment or clinical conference or catalogs or case reports).pt. |  |

mp denotes multi-purpose and implies a search through all fields; .sh. denotes a Medical Subject Heading (MeSH) term; .ti,ab. denotes a search for terms in title and abstract; exp denotes explode and implies that a term and a collection of variations on that term are searched for; * is used for truncation; ? is a single space wildcard term.

#### Motivation for selected measures to compare models

The coefficient estimate is usually a measure of secondary importance in that most applications of NMA are not used to assess the degree to which a given covariate impacts the outcome of interest. As such, it provides a secondary measure by which to assess the impact of the adjustment as well as provide a measure by which to assess the existence and extent to which a chosen covariate influences the outcome of interest. The hypothesis was that the estimates would be more precise, because more points are used to estimate them, and that they might be larger due to having better estimates of standard deviation.

The deviance information criterion (DIC) was used to compare the goodness-of-fit of competing models.^1^ DIC provides a measure of model fit that penalizes model complexity according to $DIC=\bar{D}+pD, pD=\bar{D}-\hat{D.}$ $\bar{D}$ (“Dbar”) is the posterior mean residual deviance, *pD* is the effective number of parameters, and $\hat{D}$ is the deviance evaluated at the posterior mean of the model parameters. In general, a more complex model will result in a better fit to the data, demonstrating a smaller residual deviance. The model with a better trade-off between fit and parsimony has a lower DIC. As suggested by Burnham and Anderson (1998), models receiving DIC within 1–2 of the ‘best’ deserve consideration, and 3–7 have considerably less support: these rules of thumb appear to work reasonably well for DIC. Certainly, we would like to ensure that differences are not due to Monte Carlo error: although this is straightforward for $\bar{D}$ (i.e., mean deviance), Zhu and Carlin (2000) have explored the difficulty of assessing the Monte Carlo error on DIC. As described in the statistical analysis plan, a difference of 3 or more in the DIC was considered to be meaningful.^2^

The fifth listed measure, the proportion of points above the third and fourth parabola, corresponds to points falling outside the acceptable range in leverage plots. Leverage plots are a useful tool to simultaneously assess an observation deviance (“how well the model fits a particular data point”) and leverage (“How influential an observation is with respect to model estimation”). Points outside these parabolas are points that are either too influential, too poor a fit, or too influential given its poor fit.

Finally, the SUCRA is a measure of treatment rankings that is viewed as being robust;^3^ however, much caution should be used in working with rankings as they can be affected by large variances to some treatments. This measure is included out of curiosity and because they are used by some,

#### Additional considerations

Inconsistency was assessed through the results of the inconsistency checks conducted for the aggregate data analyses. There was no evidence of inconsistency in these networks of evidence.

While previous literature has been clear on statistical models, they have been less clear on the development of some of the peripheral measures and considerations used in these models. Among these is the calculation of residual deviances in the IPD step of the model. A reminder that the overall residual deviance, $\bar{D}_{res}$, is an absolute measure of model fit, which is critical to such steps as model selection and evaluation. As shown above, it is a key component of the calculation of the DIC. Deviance for the AgD section of the model followed the methods outlined in TSD 2.^4^ Specifically, for using a binomial likelihood to model dichotomous outcomes, the equation for the total residual sum is:

| $\sum_{i} \sum_{k} 2\left( r_{ik}log\left( \frac{r_{ik}}{\hat{r}_{ik}} \right)+\left( n_{ik}-r_{ik} \right)log\left( \frac{(n_{ik}-r_{ik})}{({n_{ik}-\hat{r}}_{ik})} \right) \right)$ | Equation 3-4 |
| --- | --- |

Where $i$ is the index for the trials and $k$ is the index for the trial arms. $r_{ik}$ are the observed number of responders in the k^th^ arm of the i^th^ trial, $\hat{r}_{ik}$ are the model-predicted number of responders in the k^th^ arm of the i^th^ trial, and $n_{ik}$ are the observed number of patients in the k^th^ arm of the i^th^ trial.

In an AgD NMA model, the components of Equation 3-4 are measured in each iteration of the MCMC simulations. The challenge lay in calculating these trial-arm based measurements when the simulations were over specific patients for trials reporting IPD. To do so, vector multiplication and the *sum()* function were used to calculate each of the measures used in the AgD calculation at the study level on the basis of individual-level measures. Using JAGS was critical here as it allowed for vector multiplication in a manner akin to R. While most of these measures could be calculated outside of the JAGS program, $\hat{r}_{ik}$ (*rhat* in JAGS code) could not and therefore, further thought would be required to program this in OpenBUGS or WinBUGS.

Where ns_ipd is the number of studies reporting IPD data, nn[i,j] represents the number of patients in the j^th^ arm of the i^th^ study, pp[i,j] represents the proportion of responders in the j^th^ arm of the i^th^ study, rr[i,j] represents the number of responders in the j^th^ arm of the i^th^ study, and rhat[i,j] represents the modeled number of responders in the j^th^ arm of the i^th^ study. In doing so, we re-create the individual elements of the AgD equation at the trial level for each IPD reporting trial.

#### Model code

The code for only two of the models are presented here, namely the one-stage and two-stage IPD-AgD binomial fixed-effects models.

***One-stage IPD-AgD Binomial fixed-effects model without meta-regression adjustments***

Model{

## Part 1: IPD ####################################################

# Notes about IPD data structure:

#

# 1. Each line represents a single patient. In this manner, the treatment may equal that of the reference arm, or not.

# 2. The data are ordered as all patients in reference groups first, followed by all patients in the treatment groups second.

# This is in order to set delta to 0 in the reference group

# 3. Must have nodes in IPD be numbered 1:ntreat_ipd

#

# nobs = scalar indicating the sum of patients in IPD trials

# nref = scalar indicating the sum of patients in IPD trials in reference arm

# ns_ipd = scalar indicating the number of IPD trials

# ns_ag = scalar indicating the number of aggregate studies

# nt = scalar indicating the number of treatments in the overall network

#

# S[i] = vector indicating the study to which the i^th line belongs to (length nobs)

# refarm[i] = vector indicating the reference treatment (length nobs)

# treat[i] = vector indicating the non-reference treatment (length nobs)

# abci[i,1] = vector indicating the abacavir status for reference arm (length nobs)

# abci[i,2] = vector indicating the abacavir status for treatment arm (length nobs)

#

# treatment[k] = vector indicating the treatment in the kth ipd trial (length ns_ipd)

# reference[k] = vector indicating the reference treatment in the kth ipd trial (length ns_ipd)

# Run through regressions

for(i in 1:nobs){

ri[i] ~ dbern(pi[i])

logit(pi[i]) <- mu[S[i]] + d[treat[i]] - d[refarm[i]] + Ba*(abci[i,2] - abci[i,1]) +

Bz*(azti[i,2] - azti[i,1]) + Bo*(othi[i,2] - othi[i,1])

}

# Get deviance contribution for ipd trials

for(j in 1:nobs){

for(i in 1:ns_ipd){

ns[i,j] <- equals(S[j],i)

}

arm1[j] <- equals(treat[j], refarm[j])

arm2[j] <- 1 - equals(treat[j], refarm[j])

}

for(i in 1:ns_ipd){

nn[i,1] <- sum(ns[i,]*arm1[])

nn[i,2] <- sum(ns[i,]*arm2[])

pp[i,1] <- sum(ns[i,]*arm1[]*ri[])/nn[i,1]

pp[i,2] <- sum(ns[i,]*arm2[]*ri[])/nn[i,2]

rrb[i,1] <- sum(ns[i,]*arm1[]*ri[])

rrb[i,2] <- sum(ns[i,]*arm2[]*ri[])

rhat[i,1] <- sum(ns[i,]*arm1[]*pi[])

rhat[i,2] <- sum(ns[i,]*arm2[]*pi[])

for(k in 1:2){

dev[i,k] <- 2*(rrb[i,k] * (log(rrb[i,k])-log(rhat[i,k])) +

(nn[i,k]-rrb[i,k])*(log(nn[i,k]-rrb[i,k]) - log(nn[i,k]-rhat[i,k])))

}

resdev[i] <- sum(dev[i,1:2])

}

# Note: All IPD trials are two arm trials, so no need for multi-arm trial correction

# Part 2: Aggregate data #########################################

for(i in 1:ns_ag){

mu[(i+ns_ipd)] ~ dnorm(0,.0001)

for (k in 1:na[i]) {

r[i,k] ~ dbin(p[i,k],n[i,k])

logit(p[i,k]) <- mu[(i+ns_ipd)] + d[t[i,k]] - d[t[i,1]] +Ba*(abc[i,k] - abc[i,1])

+Bz*(azt[i,k] - azt[i,1])+Bo*(oth[i,k] - oth[i,1])+ Bt*(taf[i,k] - taf[i,1])

rhat[(i+ns_ipd),k] <- p[i,k] * n[i,k]

dev[(i+ns_ipd),k] <- 2 * (r[i,k] * (log(r[i,k])-log(rhat[(i+ns_ipd),k])) +

(n[i,k]-r[i,k]) * (log(n[i,k]-r[i,k]) - log(n[i,k]-rhat[(i+ns_ipd),k])))

}

resdev[(i+ns_ipd)] <- sum(dev[(i+ns_ipd),1:na[i]])

}

totresdev <- sum(resdev[])

for(i in 1:ns_ipd){

for (k in 2:(maxn + 1)){

dev[i,k+1] <- 0

rhat[i,k+1] <- 0

}

}

for(i in 1:ns_ag){

for (k in (na[i]):(maxn + 1)){

dev[i+ns_ipd,k+1] <- 0

rhat[(i+ns_ipd),k+1] <- 0

}

}

# Priors #####################################################

[1]<-0

for (k in 2:nt){

d[k] ~ dnorm(0,.0001)

}

for (k in 1:ns_ipd){

mu[k] ~ dnorm(0,.0001)

}

Ba ~ dnorm(0,.0001)

Bz ~ dnorm(0,.0001)

Bo ~ dnorm(0,.0001)

Bt ~ dnorm(0,.0001)

#Output ####################################################

for (c in 1:(nt-1)) {

for (k in (c+1):nt) {

OR[c,k] <- exp(d[k] - d[c])

lor[c,k] <- (d[k]-d[c])

better[c,k]<- step(lor[c,k])

RR[c,k] <-T[k]/T[c]

RD[c,k] <-T[k]-T[c]

}

}

for (c in 1:(nt)) {

for (k in 1:(c)) {

OR[c,k] <- 0

lor[c,k] <- 0

better[c,k]<- 0

RR[c,k] <-0

RD[c,k] <-0

}

}

for (i in 1: ns_ag){

mu1[i] <- mu[i]*equals(t[i,1],1)

}

A<- sum(mu1[])/nt1

for (k in 1:nt) {

logit(T[k]) <- A + d[k]

}

}

***Two-stage IPD-AgD Binomial fixed-effects model with meta-regression adjustments***

model{

for(i in 1:nobs){

ri[i] ~ dbern(pi[i])

logit(pi[i]) <- mu[S[i]] + d[treat[i]]-d[refarm[i]] + sum(beta0i[i,]) + sum(betaxi[i,])

+ Ba*(abci[i,2]-abci[i,1]) + Bz*(azti[i,2]-azti[i,1]) + Bo*(othi[i,2]-othi[i,1])

for (ii in 1:nc) {

beta0i[i,ii] <- beta0[S[i],ii]*(xi[i,ii]-center_ipd[ii])

betaxi[i,ii] <- (beta[treat[i],ii]-beta[refarm[i],ii])*(xi[i,ii]-center_ipd[ii])

}

}

for(j in 1:nobs){

for(i in 1:ns_ipd){

ns[i,j] <- equals(S[j],i)

}

arm1[j] <- equals(treat[j], refarm[j])

arm2[j] <- 1 - equals(treat[j], refarm[j])

}

for(i in 1:ns_ipd){

nn[i,1] <- sum(ns[i,]*arm1

nn[i,2] <- sum(ns[i,]*arm2[])

pp[i,1] <- sum(ns[i,]*arm1[]*ri[])/nn[i,1]

pp[i,2] <- sum(ns[i,]*arm2[]*ri[])/nn[i,2]

rrb[i,1] <- sum(ns[i,]*arm1[]*ri[])

rrb[i,2] <- sum(ns[i,]*arm2[]*ri[])

rhat[i,1] <- sum(ns[i,]*arm1[]*pi[])

rhat[i,2] <- sum(ns[i,]*arm2[]*pi[])

for(k in 1:2){

dev[i,k] <- 2*(rrb[i,k] * (log(rrb[i,k])-log(rhat[i,k])) + (nn[i,k]-

rrb[i,k])*(log(nn[i,k]-rrb[i,k]) - log(nn[i,k]-rhat[i,k])))

}

resdev[i] <- sum(dev[i,1:2])

}

# Part 2: Aggregate data ##########################################

for(i in 1:ns_ag){

mu[(i+ns_ipd)] ~ dnorm(0,.0001)

for (k in 1:na[i]) {

r[i,k] ~ dbin(p[i,k],n[i,k])

logit(p[i,k]) <- mu[(i+ns_ipd)] + d[t[i,k]] - d[t[i,1]] + sum(betax[i,k,]) + Ba*(abc[i,k] –

abc[i,1])+Bz*(azt[i,k] - azt[i,1])+Bo*(oth[i,k] - oth[i,1]) + Bt*(taf[i,k] - taf[i,1])

for (ii in 1:nc) {

betax[i,k,ii]<-(beta[t[i,k],ii]-beta[t[i,1],ii])*(x[i,ii]-center_agd[ii])

}

rhat[(i+ns_ipd),k] <- p[i,k] * n[i,k]

dev[(i+ns_ipd),k] <- 2 * (r[i,k] * (log(r[i,k])-log(rhat[(i+ns_ipd),k])) + (n[i,k]-r[i,k])*

(log(n[i,k]-r[i,k]) - log(n[i,k]-rhat[(i+ns_ipd),k])))

}

resdev[(i+ns_ipd)] <- sum(dev[(i+ns_ipd),1:na[i]])

}

totresdev <- sum(resdev[])

for(i in 1:ns_ipd){

for (k in 2:(maxn + 1)){

dev[i,k+1] <- 0

rhat[i,k+1] <- 0

}

}

for(i in 1:ns_ag){

for (k in (na[i]):(maxn + 1)){

dev[i+ns_ipd,k+1] <- 0

rhat[i+ns_ipd,k+1] <- 0

}

}

# Priors #####################################################

for(k in 1:ns_ipd){

for (ii in 1:nc) {

beta0[k,ii]<-B0[ii]

}

}

d[1]<-0

for (ii in 1:nc) {

beta[1,ii]<-0

B0[ii]~ dnorm(cov0_mean[ii],cov0_prec[ii])

}

for (k in 2:4){

d[k] ~ dnorm(0,.0001)

for (ii in 1:nc) {

beta[k,ii] ~ dnorm(cov_mean[ii, (k-1)], cov_prec[ii, (k-1)])

}

}

for (k in 5:nt){

d[k] ~ dnorm(0,.0001)

for (ii in 1:nc) {

beta[k,ii] ~ dnorm(cov_mean[ii, 1], cov_prec[ii, 1])

}

}

for (k in 1:ns_ipd){

mu[k] ~ dnorm(0,.0001)

}

Ba ~ dnorm(0,.0001)

Bz ~ dnorm(0,.0001)

Bo ~ dnorm(0,.0001)

Bt ~ dnorm(0,.0001)

#Output ####################################################

for (c in 1:(nt-1)) {

for (k in (c+1):nt) {

OR[c,k] <- exp(d[k] - d[c])

lor[c,k] <- (d[k]-d[c])

better[c,k]<- step(-lor[c,k])

RR[c,k] <-T[k]/T[c]

RD[c,k] <-T[k]-T[c]

}

}

for (c in 1:(nt)) {

for (k in 1:(c)) {

OR[c,k] <- 0

lor[c,k] <- 0

better[c,k]<- 0

RR[c,k] <-0

RD[c,k] <-0

}

}

rk <- rank(d)

for (k in 1:nt) {

best[k] <- equals(rk[k],1)

for (i in 1:nt){

prk[i,k] <- equals(rk[k],i)

}

}

for (i in 1: ns_ag){

mu1[i] <- mu[i]*equals(t[i,1],1)

}

A<- sum(mu1[])/nt1

for (k in 1:nt) {

logit(T[k]) <- A + d[k]

}

}

### Additional Results

#### Risk of bias

The following table summarizes the critical appraisals for randomized and non-randomized studies using the Cochrane Risk of Bias instrument.

Table 3: Cochrane risk of bias quality assessment for randomized controlled trials, arranged by review sub-population

| Trial | Sequence generation | Allocation concealment | Blinding | Incomplete outcome data | Selective outcome reporting | Other sources of bias |
| --- | --- | --- | --- | --- | --- | --- |
| 1439-007 Study | Unclear | Unclear | Unclear | Low | Low | Unclear |
| 2NN | Low | Low | High | Low | Low | Low |
| ACTG A5142 | Low | Low | High | Unclear | Low | Low |
| ACTG A5202 | Low | Low | High | Low | Low | Low |
| ACTG A5257 | Low | Low | High | Low | Low | Low |
| Advanz-3 | Low | Low | High | High | Low | Unclear |
| Albini et al, 2012 | Low | Low | Unclear | Low | Low | Low |
| Altair | Low | Low | High | Low | Low | Low |
| ANRS 12115 DAYANA Trial | Low | Low | High | Low | Unclear | Low |
| ARIA | Low | Low | High | Low | Low | Unclear |
| ARTEMIS | Low | Low | High | Low | Low | Low |
| ARTEN | Unclear | Low | High | Low | Low | Low |
| ASSERT | Unclear | Low | High | High | Low | Low |
| ATADAR | Low | Low | High | Low | Low | Low |
| Avihingsanon et al, 2010 | Low | Low | High | Low | Low | High |
| CASTLE | Low | Low | High | Low | Low | Low |
| CNA30024 | Unclear | Low | Low | Low | Low | Low |
| CTN177 | Low | Low | High | Low | Unclear | Low |
| DAUFIN | Unclear | Low | High | High | Low | Low |
| DRIVE AHEAD | Unclear | Unclear | Low | Unclear | Unclear | Unclear |
| DRIVE FORWARD | Unclear | Unclear | Unclear | Unclear | Unclear | Unclear |
| ECHO | Low | Low | Low | Low | Low | Low |
| ENCORE1 | Low | Low | Unclear | Low | Low | Low |
| ENCORE1 | Low | Low | Low | Low | Low | Low |
| Epzicom-Truvada | Low | Low | High | Low | Low | Low |
| FLAMINGO | Low | Low | High | Low | Low | Low |
| GESIDA 3903 | Low | Low | High | Low | Low | Low |
| GS-US-141-1475 | Low | Low | Low | Low | Low | Low |
| GS-US-236-0102 | Low | Low | Low | Low | Low | Low |
| GS-US-236-0103 | Low | Low | Low | Low | Low | Low |
| GS-US-236-0104 | Low | Low | Low | Low | Low | Low |
| GS-US-236-0140 | Unclear | Unclear | Unclear | Low | Low | Unclear |
| GS-US-292-0102 | Low | Low | Low | Low | Low | Low |
| GS-US-292-0104 | Low | Low | Low | Unclear | Low | Unclear |
| GS-US-292-0111 | Low | Low | Low | Unclear | Low | Unclear |
| GS-US-299-0102 | Low | Low | Low | Low | Low | Low |
| GS-US-380-1489 | Low | Low | Low | Low | Low | Low |
| GS-US-380-1490 | Low | Low | Low | Low | Low | Low |
| HEAT | Unclear | Low | Low | High | Low | Low |
| Lake Study | Unclear | Low | High | High | Low | Low |
| Li et al, 2008 | Unclear | Low | High | High | Low | Low |
| Maggiolo et al, 2003 | Low | Low | Low | Low | Low | Unclear |
| MASTER | Low | Low | High | Low | Unclear | High |
| METABOLIK | Unclear | Low | High | Low | Low | Low |
| NEWART | Low | Low | High | Low | Low | Low |
| OzCombo 2 | Unclear | Low | High | Low | Low | Low |
| PEARLS | Low | Low | High | Low | Low | Low |
| Phidisa II | Low | Low | High | Low | Low | Low |
| Protocol 004 | Unclear | Unclear | Low | Low | Low | Low |
| SEARCH 003 | Low | Low | High | High | Low | Low |
| SENC | Unclear | Low | High | High | Low | Low |
| Sierra-Madero et al, 2010 | Low | Low | High | High | Low | Low |
| SINGLE | Low | Low | Unclear | Low | Low | Low |
| Sinha et al, 2017 | Unclear | Low | Unclear | Unclear | Low | High |
| SPRING-1 | Low | Low | High | Low | Low | Low |
| SPRING-2 | Low | Low | Low | Low | Low | Low |
| SSAT066 | Unclear | Unclear | Unclear | High | High | Unclear |
| STaR | Low | Low | High | Low | Low | Low |
| STARTMRK | Low | Low | Low | Low | Low | Low |
| Study 903 | Low | Low | High | High | Low | Low |
| Study 934 | Low | Low | High | High | Low | Low |
| THRIVE | Low | Low | Low | Low | Low | Low |
| TMC278-C204 | Low | Low | High | Low | Low | Low |
| Tshepo | Low | Low | High | Unclear | Low | Low |
| Japanese Anti-HIV-1 QD Therapy | Unclear | Low | High | Low | Unclear | Low |
| WAVES | Low | Low | Low | Low | Low | Low |
| Zhang et al, 2015 | Unclear | Unclear | Unclear | Unclear | Unclear | Unclear |

#### Risk of bias across studies

The figures in this appendix are grouped barcharts. The bars represent the mean or median value of a characteristic for a given trial. For example, in **Figure 1** the height of each bar represents the mean age of patients in the corresponding trial. The bars are grouped according to the treatment comparisons. In a network, the treatment comparisons correspond to the lines joining the nodes (called edges). Therefore, in a two-treatment trial, there is a single comparison, but in a three-treatment trial, there are three comparisons (A-B, A-C, B-C). By combining the observations according to comparisons, two features can quickly be assessed. First, variability within a comparison can be evaluated. High variability would be indicative of heterogeneity. Second, the variability across groupings indicates imbalances across the network, which may lead to incoherence or bias. If an imbalanced factor is an effect modifier, then the imbalance is likely to lead to biased estimates. As such, these figures are used to assess potential effect modifier imbalances.

Figure 1: Comparison of baseline age across treatment comparisons


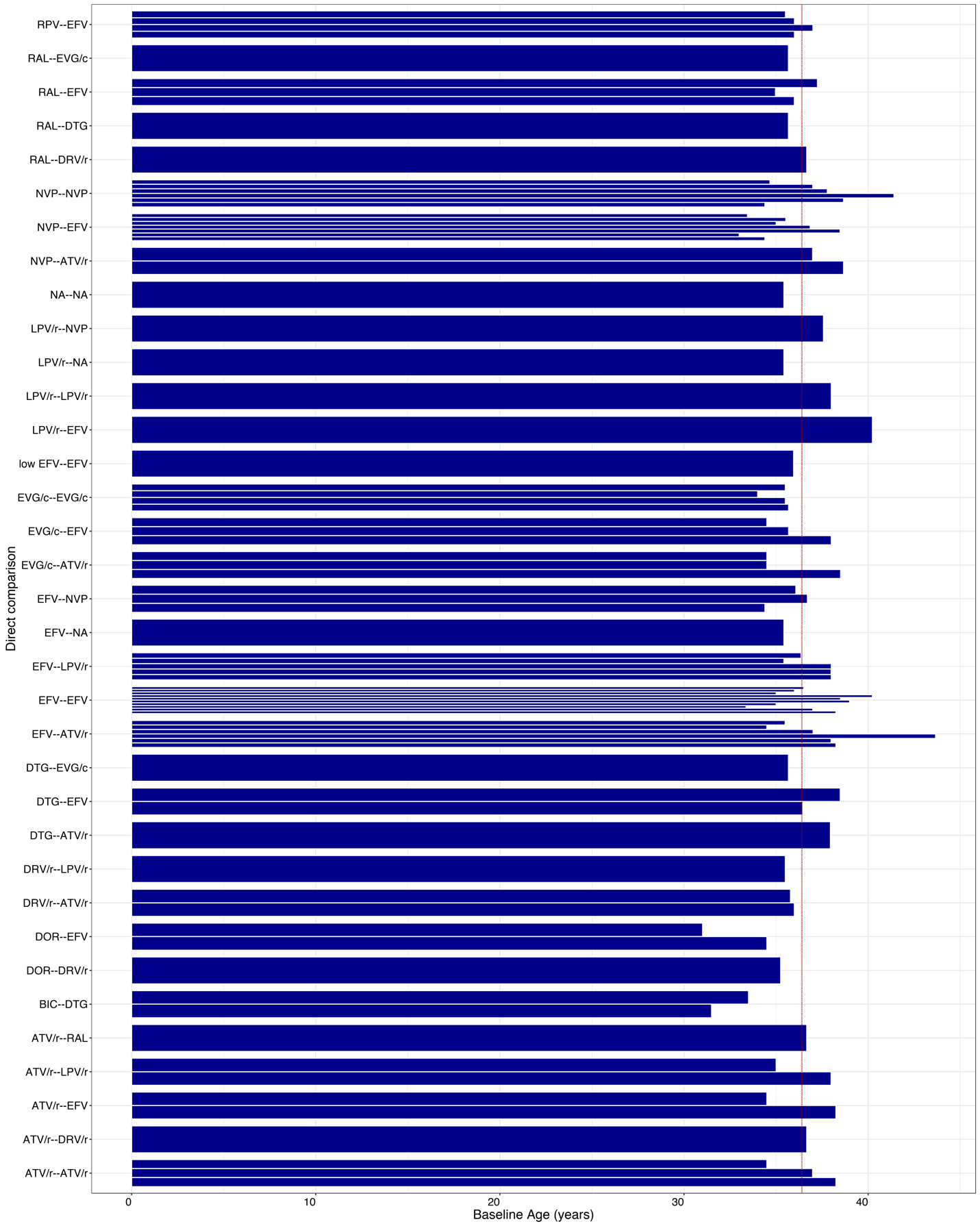


Figure 2: Comparison of percentage males across treatment comparisons


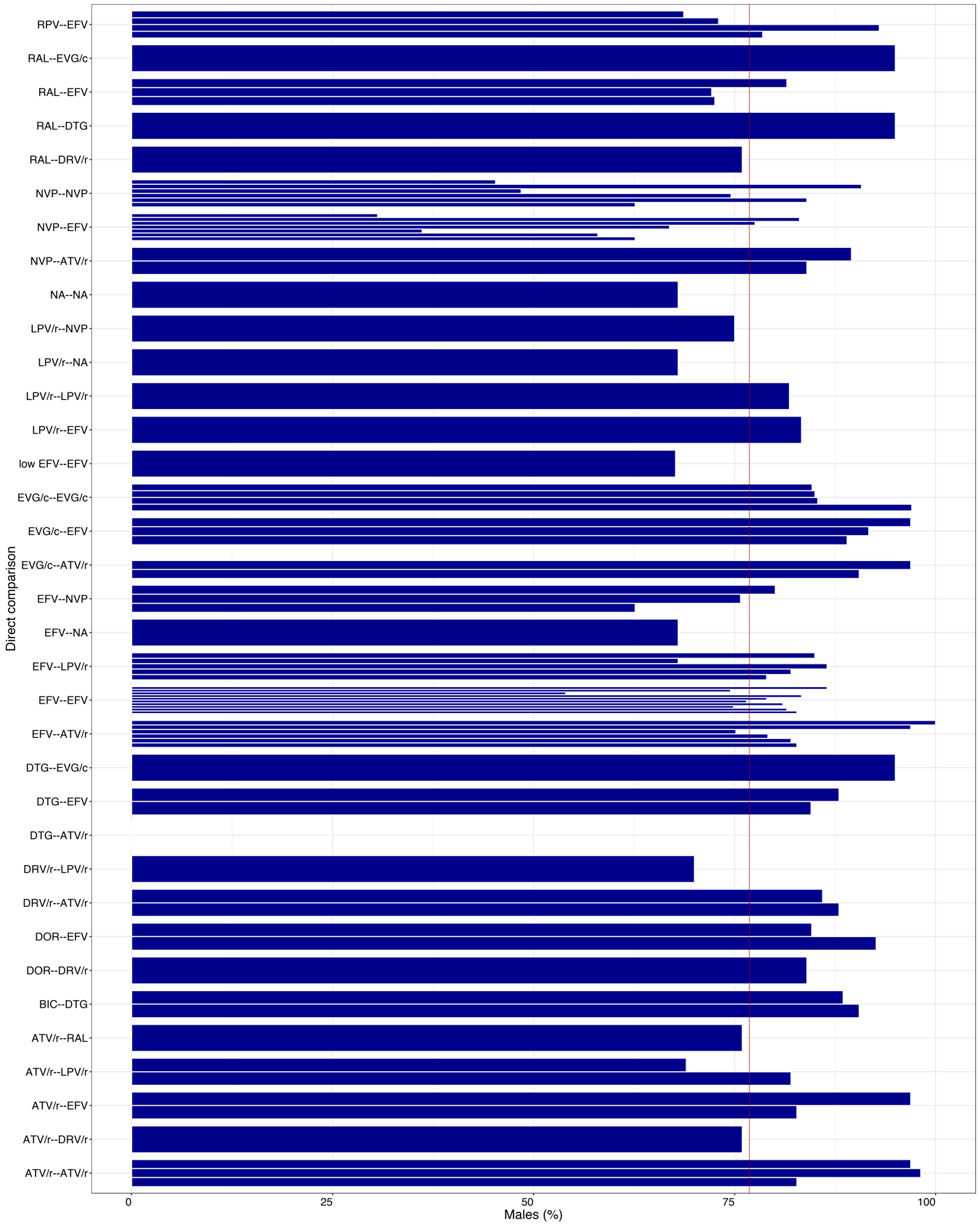


Figure 3: Comparison of baseline CD4 across treatment comparisons


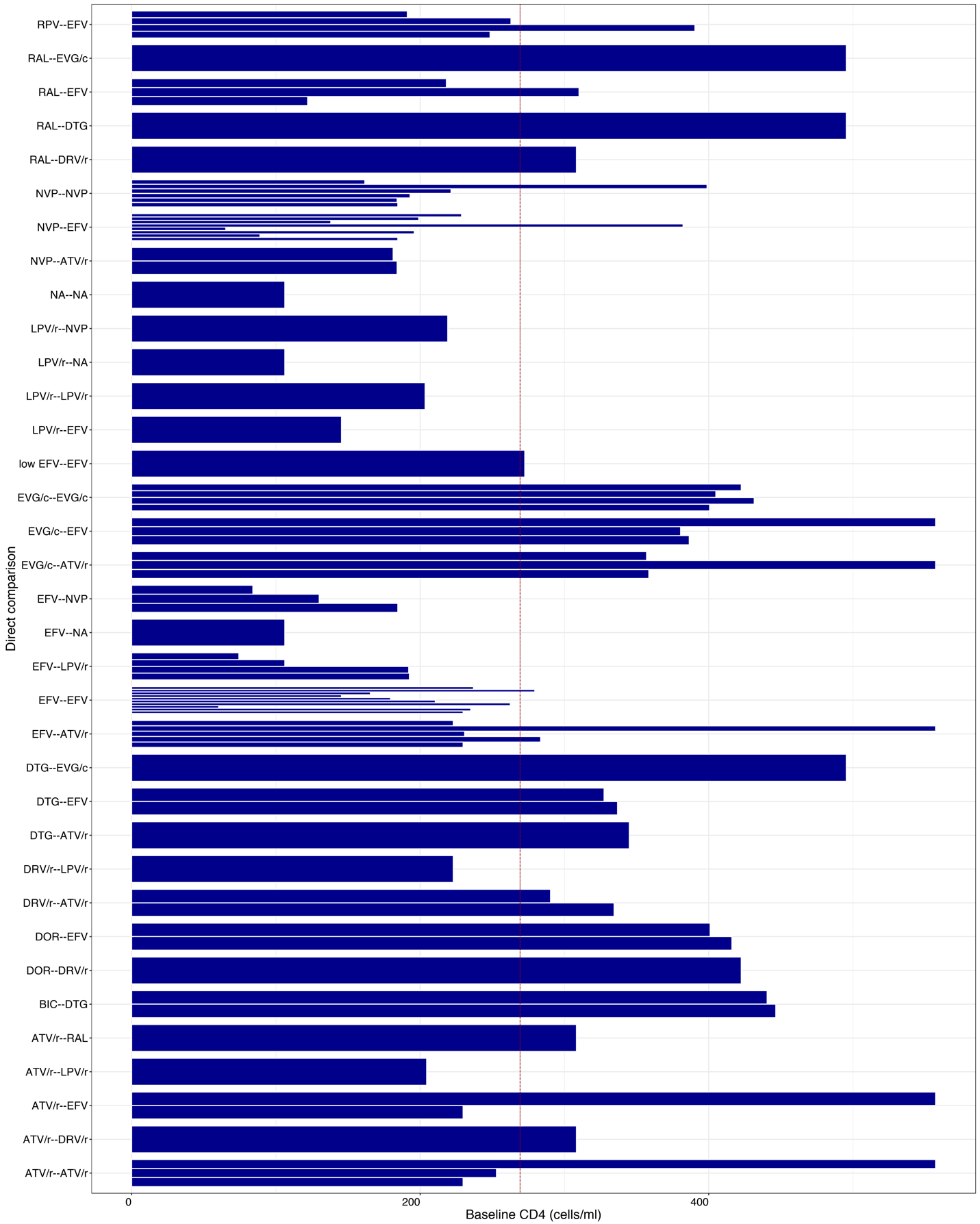


Figure 4: Comparison of baseline HIV RNA across treatment comparisons


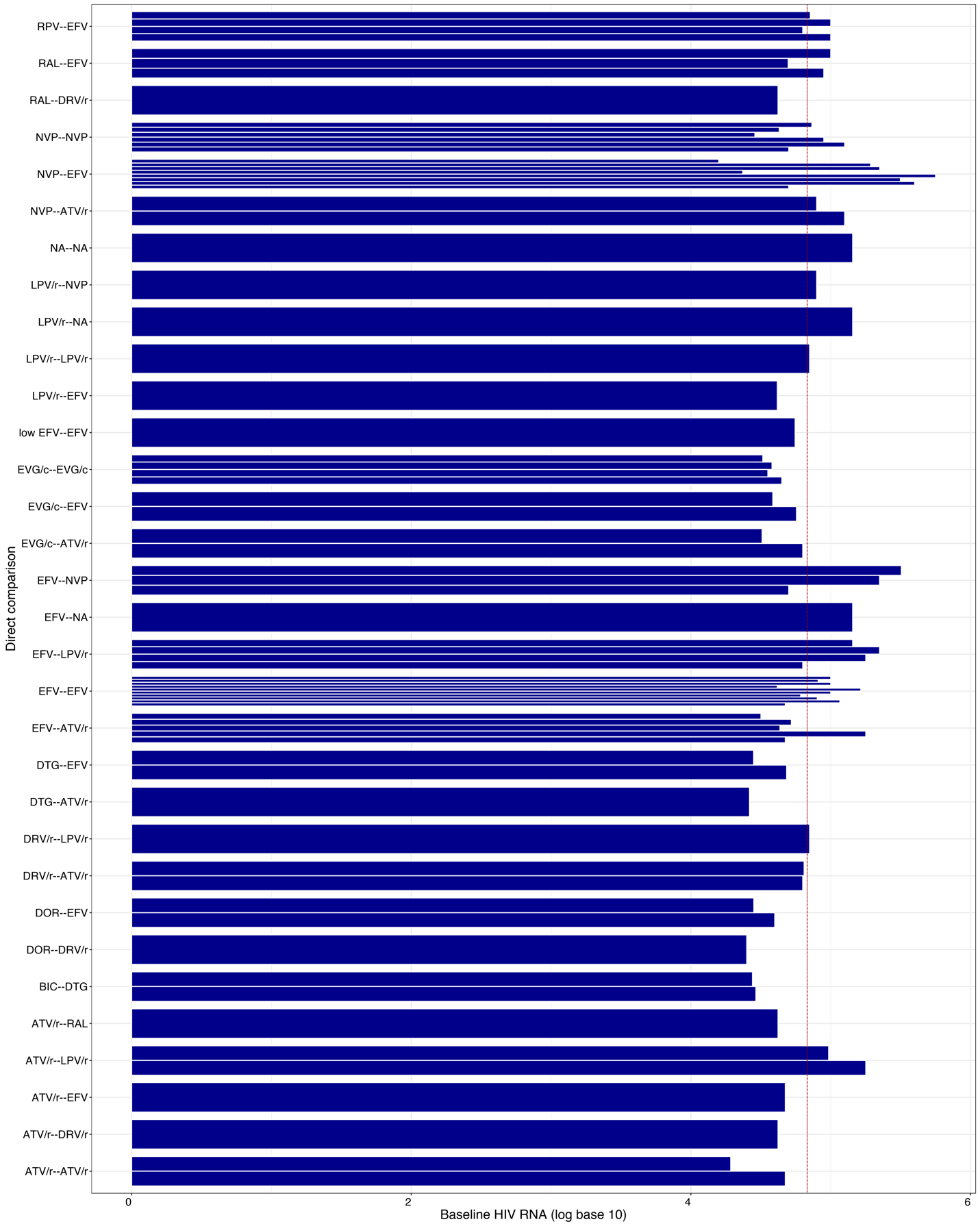


#### Results of individual studies

Table 4: Results by study arm for the primary outcomes

| Trial ID | Treatment | VS at 48w   Resp/N | Change in CD4 at 48w   Mean (SD) | Disc Resp/N | Disc due  to AEs   Resp/N |
| --- | --- | --- | --- | --- | --- |
| 1439-007 Study | DOR | 84/108 | 192(15.3) | 13/108 | 3/108 |
| 1439-007 Study | EFV | 85/108 | 195(15.3) | 16/108 | 6/108 |
| 2NN | EFV | 269/387 | 160(6.2) | 63/400 | -- |
| 2NN | NVP | 154/220 | 170(7.3) | 38/220 | -- |
| 2NN | NVP | 260/400 | 160(6.7) | 65/387 | -- |
| A5202 | ATV/r | 348/465 | 175(6.3) | 89/465 | 6/465 |
| A5202 | ATV/r | 322/463 | 177.5(5.8) | 96/463 | 4/463 |
| A5202 | EFV | 372/464 | 163(5.3) | 82/464 | 0/464 |
| A5202 | EFV | 346/465 | 188(6) | 99/465 | 4/465 |
| ACTG A5257 | ATV/r | 544/605 | 218(6.4) | 89/605 | 12/605 |
| ACTG A5257 | DRV/r | 523/601 | 201(6.9) | 101/601 | 14/601 |
| ACTG A5257 | RAL | 555/603 | 218(6.9) | 72/603 | 6/603 |
| Advanz-3 | ATV/r | 17/30 | 197(29.2) | 11/30 | 3/30 |
| Advanz-3 | EFV | 19/29 | 191(29.7) | 9/28 | 1/28 |
| Advanz-3 | LPV/r | 16/30 | 206(NA) | 14/29 | 3/29 |
| Albini et al, 2012 | ATV/r | 48/48 | 472.8(22.1) | 7/48 | 6/48 |
| Albini et al, 2012 | EFV | 43/43 | 480.3(40.7) | 10/43 | 8/43 |
| Altair | ATV/r | 93/105 | 192(15.6) | 6/105 | 5/105 |
| Altair | EFV | 97/114 | 187(15) | 7/114 | 6/114 |
| ARIA | ATV/r | 176/247 | 230.7(12.1) | 55/247 | 18/247 |
| ARIA | DTG | 203/248 | 248.8(10.9) | 42/248 | 10/248 |
| ARTEMIS | DRV/r | 288/343 | 137(21.2) | 59/343 | 13/343 |
| ARTEMIS | LPV/r | 270/346 | 141(21.2) | 81/346 | 32/346 |
| ARTEN | ATV/r | 126/193 | 186(NA) | 41/193 | 10/193 |
| ARTEN | NVP | 126/188 | 170(11.7) | 63/188 | 26/188 |
| ARTEN | NVP | 125/188 | 170(11.7) | 72/188 | 32/188 |
| ASSERT | EFV | 121/192 | 150(9.4) | 80/195 | 33/192 |
| ASSERT | EFV | 145/193 | 150(7.2) | 63/197 | 28/193 |
| ATADAR | ATV/r | -- | -- | 21/91 | 7/91 |
| ATADAR | DRV/r | -- | -- | 17/89 | 5/89 |
| CASTLE | ATV/r | 343/440 | 203(7.1) | 76/441 | 13/441 |
| CASTLE | LPV/r | 338/443 | 219(7.2) | 95/437 | 22/437 |
| CNA30024 | EFV | 226/324 | 209(14.8) | 71/324 | 16/324 |
| CNA30024 | EFV | 224/325 | 155(14.8) | 75/325 | 25/325 |
| CTN177 | LPV/r | 17/25 | 190(30.6) | 5/24 | 2/24 |
| CTN177 | NVP | 13/26 | 133.5(30.6) | 8/27 | 5/27 |
| DAYANA | EFV | 21/30 | 322(29.2) | 7/29 | 0/29 |
| DAYANA | NVP | 24/31 | 341(28.7) | 6/31 | 0/31 |
| DRIVE AHEAD | DOR | 306/364 | 198(8.6) | -- | 11/364 |
| DRIVE AHEAD | EFV | 295/364 | 188(8.8) | -- | 24/364 |
| DRIVE FORWARD | DOR | 321/383 | 193(10.7) | 56/383 | 6/383 |
| DRIVE FORWARD | DRV/r | 306/383 | 186(9.4) | 71/383 | 12/383 |
| ECHO | EFV | 285/344 | 182(8.4) | 56/344 | 27/344 |
| ECHO | RPV | 287/346 | 196(8.4) | 50/346 | 8/346 |
| ENCORE1 | EFV | 260/309 | 162(6.5) | 23/309 | 34/309 |
| ENCORE1 | EFV400 | 276/321 | 187(6.8) | 22/321 | 16/321 |
| Epzicom-Truvada | ATV/r | 44/55 | 192(21.5) | 10/55 | 6/55 |
| Epzicom-Truvada | ATV/r | 44/54 | 216(21.7) | 10/54 | 4/54 |
| FLAMINGO | DRV/r | 200/242 | 210(8.6) | 52/242 | 13/242 |
| FLAMINGO | DTG | 217/242 | 210(11) | 34/242 | 6/242 |
| GESIDA 3903 | EFV | 130/186 | 158(9.9) | 39/186 | 14/186 |
| GESIDA 3903 | EFV | 115/183 | 163(8.4) | 54/183 | 26/183 |
| GS-US-236-0102 | EFV | 296/352 | 206(8.6) | 81/352 | 26/352 |
| GS-US-236-0102 | EVG/c | 305/348 | 239(9.3) | 64/348 | 21/348 |
| GS-US-236-0103 | ATV/r | 308/355 | 211(8.5) | 55/355 | 28/355 |
| GS-US-236-0103 | EVG/c | 316/353 | 207(8.7) | 49/353 | 21/353 |
| GS-US-236-0104 | EFV | 19/23 | 166(33) | 3/23 | 1/23 |
| GS-US-236-0104 | EVG/c | 43/48 | 240(24.9) | 3/48 | 0/48 |
| GS-US-236-0140 | ATV/r | -- | -- | 3/18 | 1/16 |
| GS-US-236-0140 | ATV/r | -- | -- | 2/18 | 0/17 |
| GS-US-236-0140 | EFV | -- | -- | 3/18 | 1/16 |
| GS-US-236-0140 | EVG/c | -- | -- | 2/18 | 1/17 |
| GS-US-292-0102 | EVG/c | 99/112 | 177(13.9) | 6/113 | 4/112 |
| GS-US-292-0102 | EVG/c | 51/58 | 204(16.1) | 5/58 | 0/58 |
| GS-US-292-0104 | EVG/c | 405/435 | 235(9) | -- | 3/438 |
| GS-US-292-0104 | EVG/c | 401/432 | 221(8.9) | -- | 8/434 |
| GS-US-292-0104; GS-US-292-0111 | EVG/c | -- | -- | 130/866 | 11/866 |
| GS-US-292-0104; GS-US-292-0111 | EVG/c | -- | -- | 156/867 | 29/867 |
| GS-US-292-0111 | EVG/c | 395/431 | 225(8.5) | -- | 3/435 |
| GS-US-292-0111 | EVG/c | 385/435 | 200(8.1) | -- | 0/437 |
| GS-US-380-1489 | BIC | 290/314 | 233(10.5) | 19/314 | 0/314 |
| GS-US-380-1489 | DTG | 293/315 | 229(10.6) | 16/315 | 4/315 |
| GS-US-380-1490 | BIC | 286/320 | 180(9.3) | 28/320 | 5/320 |
| GS-US-380-1490 | DTG | 302/325 | 201(9.2) | 20/325 | 1/325 |
| HEAT | LPV/r | 232/343 | 201(6.5) | 109/343 | 20/343 |
| HEAT | LPV/r | 232/345 | 173(6.2) | 124/345 | 21/345 |
| Japanese Anti-HIV-1 QD Therapy | ATV/r | 27/35 | -- | 6/35 | 2/35 |
| Japanese Anti-HIV-1 QD Therapy | EFV | 28/36 | -- | 7/36 | 5/36 |
| Lake | EFV | 41/63 | 298(30.7) | 18/63 | 14/63 |
| Lake | LPV/r | 37/63 | 249(30.2) | 23/63 | 8/63 |
| Li et al, 2008 | NVP | -- | 367(19.6) | 19/65 | 13/65 |
| Li et al, 2008 | NVP | -- | 381(17.7) | 16/69 | 12/69 |
| Li et al, 2008 | NVP | -- | 356(20.1) | 20/64 | 12/64 |
| Maggiolo et al, 2003 | EFV | 26/34 | 194(27.4) | 5/34 | 3/34 |
| Maggiolo et al, 2003 | EFV | 26/34 | 183(27.4) | 5/34 | 4/34 |
| MASTER | EFV | -- | -- | 0/10 | -- |
| MASTER | EFV | -- | -- | 1/11 | -- |
| MASTER | LPV/r | -- | -- | 1/9 | -- |
| METABOLIK | ATV/r | 22/31 | 205.3(28.7) | 6/31 | 2/31 |
| METABOLIK | DRV/r | 26/34 | 217.4(27.4) | 5/34 | 0/34 |
| NEWART | ATV/r | 50/77 | 160.4(14.8) | 12/77 | 9/77 |
| NEWART | NVP | 46/75 | 155.1(17.5) | 19/75 | 9/75 |
| OzCombo2 | NVP | 11/20 | 172(50.1) | 4/20 | 3/20 |
| OzCombo2 | NVP | 13/22 | 201(41.4) | 6/22 | 4/22 |
| OzCombo2 | NVP | 16/23 | 190(48.2) | 7/23 | 3/23 |
| PEARLS | EFV | 437/519 | 152(4.9) | 55/519 | -- |
| PEARLS | EFV | 455/526 | 159(4.5) | -- | -- |
| PHIDISA II | EFV | -- | 148(5.4) | 50/872 | -- |
| PHIDISA II | LPV/r | -- | 165(5.4) | 37/873 | -- |
| Protocol 004 | EFV | 33/38 | 170(23) | 6/38 | 1/38 |
| Protocol 004 | RAL | 36/41 | 144(17.9) | 23/160 | 3/160 |
| SEARCH 003 | NVP | -- | -- | -- | 7/49 |
| SEARCH 003 | NVP | -- | -- | -- | 18/50 |
| SEARCH 003 | NVP | -- | -- | -- | 11/51 |
| SENC | EFV | 23/31 | 117(10.4) | 8/31 | 4/31 |
| SENC | NVP | 23/36 | 119(16.7) | 13/36 | 3/36 |
| Sierra-Madero et al, 2010 | EFV | 67/95 | 234(20) | 27/95 | 5/95 |
| Sierra-Madero et al, 2010 | LPV/r | 50/94 | 239(20) | 39/94 | 11/94 |
| SINGLE | DTG | 364/414 | 267(9.2) | 97/414 | 16/414 |
| SINGLE | EFV | 338/419 | 208(7.7) | 141/419 | 58/419 |
| SPRING-1 | DTG | 46/51 | 231(22.7) | 5/51 | 2/51 |
| SPRING-1 | EFV | 40/50 | 174(22.7) | 10/50 | 5/50 |
| SPRING-2 | DTG | 361/411 | 230(7.7) | 62/411 | 10/411 |
| SPRING-2 | RAL | 351/411 | 230(7.9) | 79/411 | 10/411 |
| STaR | EFV | 320/392 | 191(7.3) | 109/392 | 43/392 |
| STaR | RPV | 338/394 | 200(8) | 84/394 | 12/394 |
| STARTMRK | EFV | 230/282 | 163(7.7) | 71/282 | 21/282 |
| STARTMRK | RAL | 241/281 | 189(7.6) | 54/281 | 13/281 |
| Study 903 | EFV | 228/299 | -- | 82/299 | 24/299 |
| Study 903 | EFV | 240/301 | -- | 100/301 | 41/301 |
| Study 934 | EFV | 194/244 | 190(8.1) | 75/257 | 12/257 |
| Study 934 | EFV | 171/243 | 158(8.1) | 105/254 | 29/254 |
| THRIVE | EFV | 276/338 | 171(8.2) | 56/338 | 25/338 |
| THRIVE | RPV | 291/340 | 189(7.4) | 44/340 | 15/340 |
| TMC278-C204 | EFV | 72/89 | 126.5(11.1) | 24/89 | 8/89 |
| TMC278-C204 | RPV | 74/93 | 122.1(11.8) | 21/93 | 8/93 |
| Tshepo | EFV | 297/325 | 135(6.5) | -- | -- |
| Tshepo | NVP | 300/325 | 144(6.3) | -- | -- |
| WAVES | ATV/r | 231/286 | 212(10.5) | 45/286 | 19/286 |
| WAVES | EVG/c | 252/289 | 221(9.7) | 29/289 | 5/289 |

VS: viral suppression; Disc: Discontinuations; Resp: Responders; N: Sample size; ATV/r: ritonavir-boosted atazanavir; DRV/r: ritonavir-boosted darunavir; DTG: dolutegravir; EFV: efavirenz; EFV400: efavirenz 400; EVG/c: elvitegravir/cobicistat; LPV/r: ritonavir-boosted lopinavir; NVP: nevirapine; RAL: raltegravir; RPV: rilpivirine; BIC: bictegravir; DOR: doravirine

Table 5: Results by study arm for the secondary outcomes

| Trial ID | Treatment | VS at 96w   Resp/N | Change in CD4 at 96w   Mean (SD) | SAEs Resp/N | AEs   Resp/N |
| --- | --- | --- | --- | --- | --- |
| 1439-007 Study | DOR | -- | -- | 11/108 | 94/108 |
| 1439-007 Study | EFV | -- | -- | 13/108 | 96/108 |
| A5202 | ATV/r | 345/465 | 251.5(6.8) | 13/464 | 141/464 |
| A5202 | ATV/r | 317/463 | 250.3(6.8) | 21/462 | 170/462 |
| A5202 | EFV | 345/464 | 220.5(6.5) | 20/461 | 187/461 |
| A5202 | EFV | 328/465 | 250.5(7.1) | 21/461 | 147/461 |
| ACTG A5142 | EFV | -- | 230(9.9) | -- | -- |
| ACTG A5142 | LPV/r | -- | 287(12.4) | -- | -- |
| ACTG A5257 | ATV/r | 534/605 | 284(7.9) | 139/605 | 489/605 |
| ACTG A5257 | DRV/r | 537/601 | 256(7.9) | 125/601 | 390/601 |
| ACTG A5257 | RAL | 566/603 | 288(8.2) | 126/603 | 359/603 |
| Advanz-3 | ATV/r | -- | -- | 6/30 | 22/30 |
| Advanz-3 | EFV | -- | -- | 2/28 | 25/28 |
| Advanz-3 | LPV/r | -- | -- | 6/29 | 24/29 |
| Altair | ATV/r | -- | -- | 15/105 | -- |
| Altair | EFV | -- | -- | 15/114 | -- |
| ARIA | ATV/r | -- | -- | 20/247 | 197/247 |
| ARIA | DTG | -- | -- | 12/248 | 195/248 |
| ARTEMIS | DRV/r | 271/343 | 171(21.2) | 24/343 | -- |
| ARTEMIS | LPV/r | 246/346 | 188(21.2) | 42/346 | -- |
| ASSERT | EFV | 98/192 | 235(13.9) | 31/192 | -- |
| ASSERT | EFV | 113/193 | 220(8.8) | 20/193 | -- |
| ATADAR | ATV/r | 75/90 | 284(23.1) | 24/91 | 22/90 |
| ATADAR | DRV/r | 71/88 | 298(19.4) | 7/89 | 7/88 |
| CASTLE | ATV/r | 327/440 | 268(7.6) | 61/441 | 283/441 |
| CASTLE | LPV/r | 302/443 | 290(8.7) | 48/437 | 282/437 |
| CNA30024 | EFV | -- | -- | 65/324 | 226/324 |
| CNA30024 | EFV | -- | -- | 46/325 | 250/325 |
| CTN177 | LPV/r | 15/25 | 282(36.3) | -- | -- |
| CTN177 | NVP | 10/26 | 176(35.6) | -- | -- |
| DAYANA | EFV | 22/30 | 398(33.1) | -- | -- |
| DAYANA | NVP | 23/31 | 398(32.6) | -- | -- |
| DRIVE AHEAD | DOR | -- | -- | 13/364 | 301/364 |
| DRIVE AHEAD | EFV | -- | -- | 21/364 | 330/364 |
| DRIVE FORWARD | DOR | -- | -- | 19/383 | 307/383 |
| DRIVE FORWARD | DRV/r | -- | -- | 23/383 | 300/383 |
| ECHO | EFV | -- | -- | 31/344 | 317/344 |
| ECHO | RPV | -- | -- | 23/346 | 303/346 |
| ENCORE1 | EFV | 268/309 | 209(8) | 32/309 | 285/309 |
| ENCORE1 | EFV400 | 277/321 | 235(8.7) | 24/321 | 291/321 |
| Epzicom-Truvada | ATV/r | 43/55 | 236(24.5) | -- | -- |
| Epzicom-Truvada | ATV/r | 39/54 | 328(24.7) | -- | -- |
| FLAMINGO | DRV/r | 164/242 | 250(12.9) | 0/242 | 217/242 |
| FLAMINGO | DTG | 194/242 | 260(10.2) | 1/242 | 222/242 |
| GESIDA 3903 | EFV | -- | -- | 1/186 | -- |
| GESIDA 3903 | EFV | -- | -- | 3/183 | -- |
| GS-US-236-0102 | EFV | 287/352 | 273(10.1) | 69/306 | -- |
| GS-US-236-0102 | EVG/c | 293/348 | 295(11.4) | 50/311 | -- |
| GS-US-236-0103 | ATV/r | 292/355 | 261(9.6) | 62/355 | 326/355 |
| GS-US-236-0103 | EVG/c | 294/353 | 256(9.7) | 58/353 | 326/353 |
| GS-US-236-0104 | EFV | -- | -- | 1/23 | 13/23 |
| GS-US-236-0104 | EVG/c | -- | -- | 2/48 | 22/48 |
| GS-US-236-0140 | ATV/r | -- | -- | 1/16 | 14/16 |
| GS-US-236-0140 | ATV/r | -- | -- | 2/17 | 15/17 |
| GS-US-236-0140 | EFV | -- | -- | 1/16 | 14/16 |
| GS-US-236-0140 | EVG/c | -- | -- | 1/17 | 12/17 |
| GS-US-292-0102 | EVG/c | -- | -- | 11/112 | 106/112 |
| GS-US-292-0102 | EVG/c | -- | -- | 3/58 | 55/58 |
| GS-US-292-0104 | EVG/c | 388/435 | 285(10.2) | 54/435 | -- |
| GS-US-292-0104 | EVG/c | 381/432 | 271(10.6) | 47/432 | -- |
| GS-US-292-0104; GS-US-292-0111 | EVG/c | -- | -- | 121/866 | 817/866 |
| GS-US-292-0104; GS-US-292-0111 | EVG/c | -- | -- | 124/867 | 833/867 |
| GS-US-292-0111 | EVG/c | 362/431 | 274(9.6) | 43/431 | -- |
| GS-US-292-0111 | EVG/c | 358/435 | 260(9.3) | 40/435 | -- |
| GS-US-380-1489 | BIC | -- | -- | 19/314 | 265/314 |
| GS-US-380-1489 | DTG | -- | -- | 25/315 | 283/315 |
| HEAT | LPV/r | 205/343 | 250(8.4) | 42/343 | 343/343 |
| HEAT | LPV/r | 200/345 | 247(8.4) | 45/345 | 345/345 |
| Japanese Anti-HIV-1 QD Therapy | ATV/r | 24/35 | -- | -- | -- |
| Japanese Anti-HIV-1 QD Therapy | EFV | 20/36 | -- | -- | -- |
| Li et al, 2008 | NVP | -- | -- | 27/65 | -- |
| Li et al, 2008 | NVP | -- | -- | 34/69 | -- |
| Li et al, 2008 | NVP | -- | -- | 26/64 | -- |
| Maggiolo et al, 2003 | EFV | -- | -- | 3/29 | -- |
| Maggiolo et al, 2003 | EFV | -- | -- | 4/29 | -- |
| METABOLIK | ATV/r | -- | -- | 5/31 | 29/31 |
| METABOLIK | DRV/r | -- | -- | 5/34 | 31/34 |
| NEWART | ATV/r | -- | -- | 7/77 | 61/77 |
| NEWART | NVP | -- | -- | 10/75 | 50/75 |
| OzCombo2 | NVP | -- | -- | 4/20 | -- |
| OzCombo2 | NVP | -- | -- | 8/22 | -- |
| OzCombo2 | NVP | -- | -- | 7/23 | -- |
| PEARLS | EFV | -- | 216(4.9) | -- | -- |
| PEARLS | EFV | -- | 226(5.8) | -- | 521/526 |
| PHIDISA II | EFV | -- | 219(6.1) | 168/888 | -- |
| PHIDISA II | LPV/r | -- | 261(6.1) | 156/883 | -- |
| Protocol 004 | EFV | 32/38 | 232(26.8) | -- | 35/38 |
| Protocol 004 | RAL | 133/160 | 221(12.5) | -- | 36/41 |
| Sierra-Madero et al, 2010 | EFV | -- | -- | 17/95 | -- |
| Sierra-Madero et al, 2010 | LPV/r | -- | -- | 21/94 | -- |
| SINGLE | DTG | 338/422 | 325.3(10.5) | 65/414 | 266/414 |
| SINGLE | EFV | 304/422 | 281.4(10.9) | 60/419 | 333/419 |
| SPRING-1 | DTG | 45/51 | 326(23.5) | 7/51 | 43/51 |
| SPRING-1 | EFV | 36/50 | 301(35.2) | 7/50 | 41/50 |
| SPRING-2 | DTG | 332/411 | 276(8.9) | 41/411 | 251/411 |
| SPRING-2 | RAL | 314/411 | 264(8.9) | 48/411 | 259/411 |
| STaR | EFV | 284/392 | 259(9.7) | 48/392 | 392/392 |
| STaR | RPV | 307/394 | 278(9.4) | 36/394 | 394/394 |
| STARTMRK | EFV | 222/282 | 224.8(10.1) | 59/282 | 263/282 |
| STARTMRK | RAL | 228/281 | 239.6(9.7) | 57/281 | 257/281 |
| Study 903 | EFV | 217/299 | -- | 81/299 | -- |
| Study 903 | EFV | 204/301 | -- | 76/301 | -- |
| Study 934 | EFV | 155/232 | 270(12.6) | 29/257 | 163/257 |
| Study 934 | EFV | 141/231 | 237(12.6) | 35/254 | 161/254 |
| THRIVE | EFV | -- | -- | 24/338 | 241/338 |
| THRIVE | RPV | -- | -- | 22/334 | 225/334 |
| TMC278-C204 | EFV | 63/89 | 159.8(13.4) | 17/279 | 123/279 |
| TMC278-C204 | RPV | 71/93 | 145.9(12.1) | 49/279 | 67/279 |
| Tshepo | EFV | 306/325 | 225(8.2) | 49/325 | -- |
| Tshepo | NVP | 287/325 | 210(8.9) | 87/325 | -- |
| WAVES | ATV/r | -- | -- | 29/286 | -- |
| WAVES | EVG/c | -- | -- | 24/289 | -- |
| Zhang et al, 2015 | EFV | 112/122 | -- | -- | 42/122 |
| Zhang et al, 2015 | NVP | 118/128 | -- | -- | 73/128 |

VS: viral suppression; SAE: Serious adverse events; AE: Adverse events; Resp: Responders; N: Sample size; ATV/r: ritonavir-boosted atazanavir; DRV/r: ritonavir-boosted darunavir; DTG: dolutegravir; EFV: efavirenz; EFV400: efavirenz 400; EVG/c: elvitegravir/cobicistat; LPV/r: ritonavir-boosted lopinavir; NVP: nevirapine; RAL: raltegravir; RPV: rilpivirine; BIC: bictegravir; DOR: doravirine

#### Additional results from Stage 1 analyses

Table 6: Comparison of surface under the cumulative ranking (SUCRA) for viral suppression at 48 weeks

| Analyses | Model | SUCRA for EFV  Rank (SUCRA) | SUCRA for DTG  Rank (SUCRA) | SUCRA for EFV_400_  Rank (SUCRA) | Change in top 3 ranked |
| --- | --- | --- | --- | --- | --- |
| AgD NMA – Unadjusted | Fixed | 8 (0.43) | 1 (0.99) | 5 (0.59) | DTG, RAL, EVG/c |
| AgD NMA meta-regression – CD4 | Fixed | 8 (0.42) | 1 (0.99) | 5 (0.60) | None |
| AgD NMA meta-regression – HIV RNA | Fixed | 8 (0.43) | 1 (0.99) | 5 (0.59) | None |
| AgD NMA meta-regression – Male | Fixed | 8 (0.42) | 1 (0.99) | 5 (0.60) | None |
| Two-stage AgD NMA – CD4 | Fixed | 7 (0.48) | 1 (0.93) | 5 (0.66) | DTG, EVG/c, RAL |
| Two-stage AgD NMA – HIV RNA | Fixed | 7 (0.48) | 2 (0.86) | 5 (0.66) | RAL, DTG, EVG/c |
| Two-stage AgD NMA – Male | Random | 7 (0.47) | 1 (0.88) | 5 (0.62) | None |
| Two-stage AgD NMA – CD4 + HIV RNA | Fixed | 7 (0.47) | 1 (0.97) | 5 (0.64) | None |
| Two-stage AgD NMA – CD4 + Male | Fixed | 7 (0.47) | 1 (0.94) | 5 (0.64) | None |
| Two-stage AgD NMA – HIV RNA + Male | Fixed | 7 (0.47) | 1 (0.94) | 5 (0.65) | DTG, EVG/c, RAL |
| Two-stage AgD NMA – CD4 + HIV RNA + Male | Fixed | 7 (0.47) | 1 (0.92) | 5 (0.65) | None |
| One-stage IPD-AgD NMA – unadjusted | Fixed | 7 (0.46) | 1 (0.97) | 5 (0.63) | None |
| One-stage IPD-AgD NMA – CD4 | Fixed | 6 (0.49) | 1 (0.97) | 5 (0.63) | None |
| One-stage IPD-AgD NMA – HIV RNA | Fixed | 7 (0.48) | 1 (0.97) | 5 (0.62) | None |
| One-stage IPD-AgD NMA – Male | Fixed | 8 (0.46) | 1 (0.97) | 5 (0.63) | None |
| One-stage IPD-AgD NMA – CD4 + HIV RNA | Fixed | 6 (0.49) | 1 (0.97) | 5 (0.59) | None |
| One-stage IPD-AgD NMA – CD4 + Male | Fixed | 6 (0.50) | 1 (0.97) | 5 (0.63) | None |
| One-stage IPD-AgD NMA – HIV RNA + Male | Fixed | 7 (0.48) | 1 (0.97) | 5 (0.61) | None |
| One-stage IPD-AgD NMA – CD4 + HIV RNA + Male | Fixed | 6 (0.49) | 1 (0.97) | 5 (0.59) | None |
| Two-stage empirical-priors – CD4 | Fixed | 6 (0.52) | 1 (0.98) | 5 (0.63) | None |
| Two-stage empirical-priors – HIV RNA | Fixed | 7 (0.49) | 1 (0.97) | 5 (0.62) | DTG, EVG/c, RPV |
| Two-stage empirical-priors – Male | Fixed | 7 (0.46) | 1 (0.97) | 6 (0.62) | None |
| Two-stage empirical-priors – CD4 + HIV RNA | Fixed | 6 (0.53) | 1 (0.98) | 5 (0.62) | None |
| Two-stage empirical-priors – CD4 + Male | Fixed | 6 (0.54) | 1 (0.98) | 5 (0.63) | DTG, EVG/c, RAL |
| Two-stage empirical-priors – HIV RNA + Male | Fixed | 7 (0.49) | 1 (0.97) | 6 (0.62) | DTG, EVG/c, RPV |
| Two-stage empirical-priors – CD4 + HIV RNA + Male | Fixed | 6 (0.55) | 1 (0.97) | 5 (0.62) | DTG, EVG/c, RPV |

AgD: Aggregate data; IPD: Individual patient data; NMA: Network meta-analysis; EFV: Efavirenz; DTG: Dolutegravir; EFV_400_: Low-dose efavirenz; RAL: Raltegravir; EVG/c: Cobicistat-boosted elvitegravir; RPV: Rilpivirine; DOR: Doravirine.

Table 7: Comparison of model selection and fit for change in CD4 at 48 weeks

| Analyses | Model | DIC | pD | Deviance | Between study heterogeneity | prop3 | prop4 |
| --- | --- | --- | --- | --- | --- | --- | --- |
| AgD NMA – Unadjusted | Fixed | 183.63 | 67.21 | 116.42 | 7.024  (0.598, 14.032) | 5/113 | 1/113 |
| AgD NMA meta-regression – CD4 | Fixed | 183.16 | 68.15 | 115.01 | 6.967  (0.712, 14.029) | 4/113 | 1/113 |
| AgD NMA meta-regression – HIV RNA | Fixed | 185.2 | 68.14 | 117.06 | 7.05  (0.83, 13.977) | 5/113 | 1/113 |
| AgD NMA meta-regression – Male | Fixed | 183.22 | 68.03 | 115.19 | 6.943  (0.583, 14.062) | 4/113 | 1/113 |
| Two-stage AgD NMA – CD4 | Fixed | 182.25 | 67.17 | 115.08 | 5.709  (0.306, 12.769) | 5/113 | 0/113 |
| Two-stage AgD NMA – HIV RNA | Fixed | 183.55 | 67.09 | 116.46 | 6.523  (0.514, 13.787) | 5/113 | 0/113 |
| Two-stage AgD NMA – Male | Fixed | 182.07 | 67.21 | 114.86 | 5.886  (0.252, 13.052) | 5/113 | 0/113 |
| Two-stage AgD NMA – CD4 + HIV RNA | Fixed | 183.76 | 67.12 | 116.64 | 6.67  (0.784, 13.806) | 5/113 | 0/113 |
| Two-stage AgD NMA – CD4 + Male | Fixed | 183.91 | 67.13 | 116.78 | 6.828  (0.619, 13.966) | 5/113 | 0/113 |
| Two-stage AgD NMA – HIV RNA + Male | Fixed | **182.02** | **67.07** | 114.95 | 5.774  (0.405, 12.739) | 5/113 | 0/113 |
| Two-stage AgD NMA – CD4 + HIV RNA + Male | Fixed | 184.23 | 67.16 | 117.07 | 6.898  (0.702, 14.065) | 5/113 | 0/113 |
| One-stage IPD-AgD NMA – unadjusted | Random | 182.07 | 83.11 | 98.96 | 12.435  (7.593, 18.689) | 5/113 | 2/113 |
| One-stage IPD-AgD NMA – CD4 | Random | 183.62 | 84.57 | 99.05 | 12.726  (7.705, 19.236) | 4/113 | 2/113 |
| One-stage IPD-AgD NMA – HIV RNA | Random | 190.39 | 86.68 | 103.71 | 14.303  (9.266, 21.299) | 5/113 | 3/113 |
| One-stage IPD-AgD NMA – Male | Random | 183.07 | 84.25 | **98.82** | 12.351  (7.511, 18.675) | 5/113 | 2/113 |
| One-stage IPD-AgD NMA – CD4 + HIV RNA | Random | 184.69 | 85.52 | 99.17 | 12.618  (7.612, 19.172) | 4/113 | 2/113 |
| One-stage IPD-AgD NMA – CD4 + Male | Random | 189.33 | 86.44 | 102.89 | 13.428  (8.301, 20.385) | 5/113 | 3/113 |
| One-stage IPD-AgD NMA – HIV RNA + Male | Random | 191.41 | 87.67 | 103.74 | 14.454  (9.218, 21.522) | 5/113 | 3/113 |
| One-stage IPD-AgD NMA – CD4 + HIV RNA + Male | Random | 190.24 | 87.41 | 102.83 | 13.472  (8.31, 20.484) | 5/113 | 3/113 |
| Two-stage empirical-priors – CD4 | Random | 183.7 | 84.71 | 98.99 | 10.86  (5.346, 17.25) | 5/113 | 2/113 |
| Two-stage empirical-priors – HIV RNA | Random | 187.76 | 87.75 | 100.01 | 13.885  (8.917, 20.646) | 4/113 | 2/113 |
| Two-stage empirical-priors – Male | Random | 185.89 | 86.69 | 99.2 | 12.489  (7.451, 19.181) | 5/113 | 2/113 |
| Two-stage empirical-priors – CD4 + HIV RNA + Male | Random | 192.44 | 92.32 | 100.12 | 12.241  (6.984, 18.916) | 5/113 | 2/113 |
| Two-stage empirical-priors – CD4 + Male | Random | 188.26 | 88.6 | 99.66 | 11.751  (6.641, 18.425) | 5/113 | 2/113 |
| Two-stage empirical-priors – HIV RNA + Male | Random | 191.92 | 91.49 | 100.43 | 14.251  (9.181, 21.108) | 4/113 | 2/113 |
| Two-stage empirical-priors – CD4 + HIV RNA | Random | 187.18 | 88.06 | 99.12 | 11.172  (5.389, 17.77) | 5/113 | 2/113 |

AgD: Aggregate data; IPD: Individual patient data; NMA: Network meta-analysis; DIC: Deviance information criterion; pD: Effective number of parameters; prop3: Proportion of observations above deviance2 + leverage = 3; prop4: Proportion of observations above deviance2 + leverage = 4. Between-study heterogeneity obtained through the random-effects model, not the fixed-effect model if it was selected.

Table 8: Comparison of surface under the cumulative ranking (SUCRA) for change in CD4 at 48 weeks

| Analyses | Model | SUCRA for EFV  Rank | SUCRA for DTG  Rank | SUCRA for EFV_400_  Rank | Change in top 3 ranked |
| --- | --- | --- | --- | --- | --- |
| AgD NMA – Unadjusted | Fixed | 12 (0.06) | 1 (0.81) | 10 (0.18) | DTG, RAL, LPV/r |
| AgD NMA meta-regression – CD4 | Fixed | 12 (0.07) | 3 (0.73) | 2 (0.79) | LPV/r, EFV_400_, DTG |
| AgD NMA meta-regression – HIV RNA | Fixed | 12 (0.06) | 2 (0.77) | 1 (0.82) | EFV_400_, DTG, EVG/c |
| AgD NMA meta-regression – Male | Fixed | 12 (0.06) | 3 (0.73) | 1 (0.90) | EFV_400_, LPV/r, DTG |
| Two-stage AgD NMA – CD4 | Fixed | 11 (0.20) | 9 (0.25) | 1 (0.91) | EFV_400_, LPV/r, EVG/c |
| Two-stage AgD NMA – HIV RNA | Fixed | 11 (0.19) | 9 (0.26) | 1 (0.90) | EFV_400_, LPV/r, EVG/c |
| Two-stage AgD NMA – Male | Fixed | 10 (0.19) | 9 (0.28) | 1 (0.90) | EFV_400_, LPV/r, EVG/c |
| Two-stage AgD NMA – CD4 + HIV RNA | Fixed | 11 (0.19) | 9 (0.26) | 1 (0.90) | EFV_400_, LPV/r, EVG/c |
| Two-stage AgD NMA – CD4 + Male | Fixed | 11 (0.19) | 9 (0.25) | 1 (0.90) | EFV_400_, LPV/r, EVG/c |
| Two-stage AgD NMA – HIV RNA + Male | Fixed | 10 (0.19) | 9 (0.29) | 1 (0.90) | EFV_400_, LPV/r, EVG/c |
| Two-stage AgD NMA – CD4 + HIV RNA + Male | Fixed | 11 (0.19) | 9 (0.25) | 1 (0.90) | EFV_400_, LPV/r, EVG/c |
| One-stage IPD-AgD NMA – unadjusted | Random | 12 (0.09) | 1 (0.86) | 5 (0.65) | DTG, RAL, BIC |
| One-stage IPD-AgD NMA – CD4 | Random | 12 (0.09) | 1 (0.87) | 4 (0.65) | DTG, RAL, EVG/c |
| One-stage IPD-AgD NMA – HIV RNA | Random | 12 (0.07) | 1 (0.89) | 4 (0.67) | DTG, RAL, EVG/c |
| One-stage IPD-AgD NMA – Male | Random | 12 (0.09) | 1 (0.87) | 4 (0.65) | DTG, RAL, EVG/c |
| One-stage IPD-AgD NMA – CD4 + HIV RNA | Random | 12 (0.09) | 1 (0.87) | 4 (0.65) | DTG, RAL, EVG/c |
| One-stage IPD-AgD NMA – CD4 + Male | Random | 12 (0.06) | 1 (0.89) | 4 (0.68) | DTG, RAL, EVG/c |
| One-stage IPD-AgD NMA – HIV RNA + Male | Random | 12 (0.07) | 1 (0.89) | 4 (0.67) | DTG, RAL, EVG/c |
| One-stage IPD-AgD NMA – CD4 + HIV RNA + Male | Random | 12 (0.06) | 1 (0.89) | 4 (0.68) | DTG, RAL, EVG/c |
| Two-stage empirical-priors – CD4 | Random | 12 (0.11) | 1 (0.92) | 5 (0.62) | None |
| Two-stage empirical-priors – HIV RNA | Random | 12 (0.08) | 1 (0.87) | 4 (0.63) | DTG, RAL, EVG/c |
| Two-stage empirical-priors – Male | Random | 12 (0.10) | 1 (0.88) | 5 (0.63) | DTG, RAL, EVG/c |
| Two-stage empirical-priors – CD4 + HIV RNA | Random | 12 (0.12) | 1 (0.93) | 5 (0.60) | None |
| Two-stage empirical-priors – CD4 + Male | Random | 12 (0.10) | 1 (0.90) | 5 (0.60) | None |
| Two-stage empirical-priors – HIV RNA + Male | Random | 12 (0.09) | 1 (0.87) | 5 (0.61) | DTG, RAL, EVG/c |
| Two-stage empirical-priors – CD4 + HIV RNA + Male | Random | 12 (0.11) | 1 (0.91) | 6 (0.59) | DTG, RAL, DRV/r |

AgD: Aggregate data; IPD: Individual patient data; NMA: Network meta-analysis; EFV: Efavirenz; DTG: Dolutegravir; EFV_400_: Low-dose efavirenz; RAL: Raltegravir; EVG/c: Cobicistat-boosted elvitegravir; RPV: Rilpivirine; DOR: Doravirine.

Table 9: Comparison of comparative treatment estimates for change in CD4 at 48 weeks

| Analyses | Model | DTG vs. EFV  MD (95% CrI) | EFV_400_ vs. EFV  MD (95% CrI) | DTG vs. EFV_400_  MD (95% CrI) | Mean change in mean change | Maximum change in mean change |
| --- | --- | --- | --- | --- | --- | --- |
| AgD NMA – Unadjusted | Fixed | 25.40 (7.11, 43.74) | 6.41 (-7.93, 20.67) | 19.03 (-4.38, 42.38) | -- | -- |
| AgD NMA meta-regression – CD4 | Fixed | 22.13 (7.24, 36.76) | 25.83 (7.52, 44.13) | -3.66 (-27.40, 20.03) | 5.621 | 19.424 |
| AgD NMA meta-regression – HIV RNA | Fixed | 22.32 (7.70, 36.76) | 25.59 (7.31, 43.87) | -3.31 (-26.62, 20.13) | 4.235 | 19.182 |
| AgD NMA meta-regression – Male | Fixed | 20.72 (6.25, 35.51) | 31.03 (10.94, 50.92) | -10.28 (-35.77, 15.29) | 5.453 | 24.623 |
| Two-stage AgD NMA – CD4 | Fixed | 0.29 (-11.96, 12.08) | 25.46 (7.41, 43.74) | -25.22 (-46.91, -3.49) | 9.556 | 25.107 |
| Two-stage AgD NMA – HIV RNA | Fixed | 0.80 (-14.01, 15.32) | 25.35 (6.99, 43.48) | -24.64 (-48.26, -1.19) | 9.332 | 24.601 |
| Two-stage AgD NMA – Male | Fixed | 1.10 (-11.20, 13.07) | 25.57 (7.37, 43.69) | -24.42 (-46.33, -2.70) | 9.386 | 24.292 |
| Two-stage AgD NMA – CD4 + HIV RNA | Fixed | 0.92 (-13.85, 15.51) | 25.44 (7.32, 43.76) | -24.57 (-48.09, -1.49) | 9.315 | 24.478 |
| Two-stage AgD NMA – CD4 + Male | Fixed | 0.77 (-13.94, 15.63) | 25.47 (7.35, 44.01) | -24.85 (-48.38, -1.38) | 9.318 | 24.627 |
| Two-stage AgD NMA – HIV RNA + Male | Fixed | 1.74 (-10.75, 13.75) | 25.58 (6.98, 43.93) | -23.92 (-45.83, -1.84) | 9.269 | 23.652 |
| Two-stage AgD NMA – CD4 + HIV RNA + Male | Fixed | 0.48 (-14.18, 15.19) | 25.50 (6.94, 43.61) | -24.91 (-48.20, -1.13) | 9.392 | 24.915 |
| One-stage IPD-AgD NMA – unadjusted | Random | 34.20 (15.99, 51.87) | 25.13 (-6.26, 56.17) | 9.15 (-26.81, 44.90) | 6.985 | 18.717 |
| One-stage IPD-AgD NMA – CD4 | Random | 34.83 (16.36, 53.34) | 25.16 (-6.63, 56.73) | 9.71 (-26.75, 46.13) | 6.837 | 18.753 |
| One-stage IPD-AgD NMA – HIV RNA | Random | 42.01 (22.23, 61.92) | 31.53 (-2.72, 65.44) | 10.45 (-28.87, 50.29) | 10.847 | 25.125 |
| One-stage IPD-AgD NMA – Male | Random | 34.56 (16.45, 52.64) | 24.95 (-6.54, 56.07) | 9.61 (-26.27, 46.10) | 6.772 | 18.537 |
| One-stage IPD-AgD NMA – CD4 + HIV RNA | Random | 34.93 (16.41, 53.59) | 24.99 (-6.51, 56.45) | 9.97 (-26.64, 46.52) | 6.91 | 18.582 |
| One-stage IPD-AgD NMA – CD4 + Male | Random | 40.90 (21.81, 60.06) | 31.50 (-1.53, 64.27) | 9.36 (-28.18, 47.70) | 10.325 | 25.094 |
| One-stage IPD-AgD NMA – HIV RNA + Male | Random | 42.15 (22.25, 62.44) | 32.06 (-2.75, 66.57) | 10.11 (-29.44, 50.44) | 10.983 | 25.647 |
| One-stage IPD-AgD NMA – CD4 + HIV RNA + Male | Random | 41.03 (21.92, 60.52) | 31.25 (-1.89, 63.95) | 9.78 (-27.94, 48.39) | 10.423 | 24.841 |
| Two-stage empirical-priors – CD4 | Random | 39.63 (21.49, 57.21) | 23.49 (-5.67, 52.61) | 16.13 (-18.02, 49.88) | 7.074 | 17.081 |
| Two-stage empirical-priors – HIV RNA | Random | 39.35 (19.62, 59.28) | 27.80 (-6.06, 61.44) | 11.57 (-27.48, 51.09) | 7.71 | 21.393 |
| Two-stage empirical-priors – Male | Random | 36.48 (17.21, 55.01) | 24.23 (-7.24, 56.09) | 12.11 (-24.56, 48.57) | 7.471 | 17.82 |
| Two-stage empirical-priors – CD4 + HIV RNA | Random | 41.04 (22.05, 59.22) | 22.57 (-7.51, 52.24) | 18.44 (-16.67, 53.23) | 7.353 | 16.163 |
| Two-stage empirical-priors – CD4 + Male | Random | 43.76 (25.08, 62.60) | 26.96 (-3.88, 57.76) | 16.83 (-19.18, 52.98) | 9.175 | 20.547 |
| Two-stage empirical-priors – HIV RNA + Male | Random | 40.48 (19.85, 61.29) | 27.36 (-7.52, 61.79) | 13.17 (-26.77, 53.69) | 8.332 | 20.949 |
| Two-stage empirical-priors – CD4 + HIV RNA + Male | Random | 44.73 (24.74, 64.68) | 26.29 (-5.56, 58.20) | 18.40 (-19.22, 55.58) | 9.283 | 19.881 |

AgD: Aggregate data; IPD: Individual patient data; NMA: Network meta-analysis; EFV: Efavirenz; DTG: Dolutegravir; EFV_400_: Low-dose efavirenz; OR: Odds ratio; CrI: Credible interval.

**Table 10: Coefficient estimates for change from baseline in CD4 cell counts at 48 weeks**

| Analyses | Model | ß_1,1_  Median  (95% CrI) | ß_1,2_  Median  (95% CrI) | ß_1,3_  Median  (95% CrI) | ß_0,1_  Median  (95% CrI) | ß_0,2_  Median  (95% CrI) | ß_0,3_  Median  (95% CrI) |
| --- | --- | --- | --- | --- | --- | --- | --- |
| AgD NMA meta-regression – CD4 | Fixed | 6.64  (-1.164, 14.47) | -- | -- | -- | -- | -- |
| AgD NMA meta-regression – HIV RNA | Fixed | 2.507  (-21.233, 26.667) | -- | -- | -- | -- | -- |
| AgD NMA meta-regression – Male | Fixed | 38.477  (-20.112, 96.806) | -- | -- | -- | -- | -- |
| One-stage IPD-AgD NMA – CD4 | Fixed | -1.435  (-7.127, 4.16) | -- | -- | 9.734  (-30.631, 46.638) | -- | -- |
| One-stage IPD-AgD NMA – HIV RNA | Fixed | **45.509**  **(31.324, 59.931)** | -- | -- | -75.763  (-149.13, 14.175) | -- | -- |
| One-stage IPD-AgD NMA – Male | Fixed | -4.014  (-30.045, 22.104) | -- | -- | -126.62  (-238.199, -0.712) | -- | -- |
| One-stage IPD-AgD NMA – CD4 + HIV RNA | Fixed | -1.371  (-7.12, 4.302) | -3.184  (-29.473, 22.936) | -- | -6.799  (-52.123, 42.331) | -140.074  (-283.332, 11.386) | -- |
| One-stage IPD-AgD NMA – CD4 + Male | Fixed | 4.659  (-1.443, 10.537) | **48.957**  **(34.006, 63.896)** | -- | -42.438  (-96.027, 25.699) | -148.536  (-246.03, -20.194) | -- |
| One-stage IPD-AgD NMA – HIV RNA + Male | Fixed | **45.738**  **(31.58, 60.273)** | 0.129  (-26.576, 26.501) | -- | -44.318  (-163.782, 53.061) | -68.567  (-252.042, 114.25) | -- |
| One-stage IPD-AgD NMA – CD4 + HIV RNA + Male | Fixed | 4.664  (-1.333, 10.613) | **48.976**  **(34.079, 63.834)** | -1.083  (-27.506, 25.397) | -47.713  (-101.172, 9.881) | -112.852  (-241.15, 7.615) | -76.08  (-233.67, 106.33) |

*Covariates 1-3 represents the covariates as ordered in the descriptor. E.g., in the last row 1 represents CD4, 2 represents HIV RNA and 3 represents male.*

Table 11: Comparison of model selection and fit for discontinuations

| Analyses | Model | DIC | pD | Deviance | Between study heterogeneity | prop3 | prop4 |
| --- | --- | --- | --- | --- | --- | --- | --- |
| AgD NMA – Unadjusted | Random | 202.25 | 80.81 | 121.44 | 0.149 (0.022, 0.288) | 2/122 | 2/122 |
| AgD NMA meta-regression – CD4 | Random | 202.61 | 81.2 | 121.41 | 0.147 (0.009, 0.29) | 2/122 | 2/122 |
| AgD NMA meta-regression – HIV RNA | Fixed | 202.38 | **70.81** | 131.57 | 0.152 (0.022, 0.29) | 5/122 | 3/122 |
| AgD NMA meta-regression – Male | Random | 200.74 | 79.17 | 121.57 | 0.148 (0.005, 0.29) | 2/122 | 2/122 |
| Two-stage AgD NMA – CD4 | Random | 202.89 | 85.55 | 117.34 | 0.222 (0.111, 0.354) | 2/122 | 1/122 |
| Two-stage AgD NMA – HIV RNA | Random | 202.90 | 85.41 | 117.49 | 0.219 (0.107, 0.35) | 2/122 | 1/122 |
| Two-stage AgD NMA – Male | Random | 207.00 | 90.70 | 116.30 | 0.279 (0.175, 0.413) | 1/122 | 1/122 |
| Two-stage AgD NMA – CD4 + HIV RNA | Random | 206.88 | 88.48 | 118.4 | 0.251 (0.142, 0.388) | 1/122 | 1/122 |
| Two-stage AgD NMA – CD4 + Male | Random | 203.46 | 86.73 | **116.73** | 0.226 (0.118, 0.357) | 2/122 | 1/122 |
| Two-stage AgD NMA – HIV RNA + Male | Random | 203.88 | 86.62 | 117.26 | 0.222 (0.111, 0.355) | 2/122 | 1/122 |
| Two-stage AgD NMA – CD4 + HIV RNA + Male | Random | 204.65 | 87.14 | 117.51 | 0.231 (0.122, 0.365) | 2/122 | 1/122 |
| One-stage IPD-AgD NMA – unadjusted | Random | 203.25 | 81.68 | 121.57 | 0.154 (0.023, 0.29) | 2/122 | 2/122 |
| One-stage IPD-AgD NMA – CD4 | Random | 205.60 | 84.36 | 121.24 | 0.18 (0.021, 0.327) | 2/122 | 2/122 |
| One-stage IPD-AgD NMA – HIV RNA | Fixed | 205.81 | 70.85 | 134.96 | 0.16 (0.012, 0.305) | 6/122 | 3/122 |
| One-stage IPD-AgD NMA – Male | Random | 204.42 | 82.82 | 121.6 | 0.163 (0.015, 0.31) | 2/122 | 2/122 |
| One-stage IPD-AgD NMA – CD4 + HIV RNA | Random | 206.51 | 85.68 | 120.83 | 0.188 (0.029, 0.337) | 2/122 | 1/122 |
| One-stage IPD-AgD NMA – CD4 + Male | Random | 206.21 | 85.83 | 120.38 | 0.186 (0.046, 0.336) | 2/122 | 1/122 |
| One-stage IPD-AgD NMA – HIV RNA + Male | Fixed | 206.94 | 71.78 | 135.16 | 0.164 (0.009, 0.32) | 6/122 | 3/122 |
| One-stage IPD-AgD NMA – CD4 + HIV RNA + Male | Fixed | 208.85 | 72.82 | 136.03 | 0.182 (0.016, 0.341) | 6/122 | 5/122 |
| Two-stage empirical-priors – CD4 | Random | 207.96 | 86.77 | 121.19 | 0.192 (0.05, 0.333) | 2/122 | 1/122 |
| Two-stage empirical-priors – HIV RNA | Fixed | 202.64 | 72.30 | 130.34 | 0.141 (0.018, 0.281) | 5/122 | 3/122 |
| Two-stage empirical-priors – Male | Fixed | 206.09 | 72.95 | 133.14 | 0.146 (0.003, 0.288) | 8/122 | 3/122 |
| Two-stage empirical-priors – CD4 + HIV RNA + Male | Fixed | 208.52 | 78.34 | 130.18 | 0.15 (0.008, 0.298) | 3/122 | 3/122 |
| Two-stage empirical-priors – CD4 + Male | Random | 206.83 | 87.04 | 119.79 | 0.174 (0.024, 0.317) | 3/122 | 1/122 |
| Two-stage empirical-priors – HIV RNA + Male | Fixed | 201.93 | 75.11 | 126.82 | 0.122 (0.011, 0.267) | 4/122 | 3/122 |
| Two-stage empirical-priors – CD4 + HIV RNA | Random | 209.80 | 88.34 | 121.46 | 0.177 (0.028, 0.323) | 2/122 | 2/122 |
| HMR IPD-AgD NMA – CD4 | Random | 205.14 | 83.4 | 121.74 | 0.157 (0.022, 0.298) | 2/122 | 2/122 |
| HMR IPD-AgD NMA – HIV RNA | Random | 202.93 | 82.28 | 120.65 | 0.148 (0.023, 0.285) | 2/122 | 2/122 |
| HMR IPD-AgD NMA – Male | Fixed | **200.41** | 71.71 | 128.7 | 0.128 (0.016, 0.269) | 4/122 | 2/122 |

AgD: Aggregate data; IPD: Individual patient data; NMA: Network meta-analysis; DIC: Deviance information criterion; pD: Effective number of parameters; prop3: Proportion of observations above deviance2 + leverage = 3; prop4: Proportion of observations above deviance2 + leverage = 4. Between-study heterogeneity obtained through the random-effects model, not the fixed-effect model if it was selected.

Table 12: Comparison of surface under the cumulative ranking (SUCRA) for discontinuations

| Analyses | Model | SUCRA for EFV  Rank (SUCRA) | SUCRA for DTG  Rank (SUCRA) | SUCRA for EFV_400_  Rank (SUCRA) | Change in top 3 ranked |
| --- | --- | --- | --- | --- | --- |
| AgD NMA – Unadjusted | Random | 10 (0.29) | 1 (0.97) | 7 (0.45) | DTG, EVG/c, RAL |
| AgD NMA meta-regression – CD4 | Random | 10 (0.32) | 1 (0.95) | 7 (0.46) | None |
| AgD NMA meta-regression – HIV RNA | Fixed | 10 (0.27) | 1 (0.96) | 7 (0.47) | None |
| AgD NMA meta-regression – Male | Random | 10 (0.34) | 1 (0.97) | 9 (0.35) | None |
| Two-stage AgD NMA – CD4 | Random | 10 (0.36) | 1 (0.95) | 7 (0.49) | DTG, EVG/c, RPV |
| Two-stage AgD NMA – HIV RNA | Random | 10 (0.36) | 1 (0.95) | 7 (0.49) | DTG, EVG/c, RPV |
| Two-stage AgD NMA – Male | Random | 10 (0.33) | 1 (0.91) | 6 (0.48) | DTG, EVG/c, DOR |
| Two-stage AgD NMA – CD4 + HIV RNA | Random | 10 (0.35) | 1 (0.90) | 7 (0.49) | DTG, EVG/c, DOR |
| Two-stage AgD NMA – CD4 + Male | Random | 10 (0.37) | 1 (0.93) | 6 (0.49) | DTG, EVG/c, DOR |
| Two-stage AgD NMA – HIV RNA + Male | Random | 10 (0.35) | 1 (0.94) | 6 (0.49) | DTG, EVG/c, DOR |
| Two-stage AgD NMA – CD4 + HIV RNA + Male | Random | 10 (0.35) | 1 (0.92) | 6 (0.49) | DTG, EVG/c, DOR |
| One-stage IPD-AgD NMA – unadjusted | Random | 10 (0.34) | 1 (0.90) | 6 (0.49) | None |
| One-stage IPD-AgD NMA – CD4 | Random | 10 (0.35) | 1 (0.90) | 6 (0.49) | DTG, RAL, EVG/c |
| One-stage IPD-AgD NMA – HIV RNA | Fixed | 10 (0.32) | 1 (0.92) | 6 (0.48) | None |
| One-stage IPD-AgD NMA – Male | Random | 10 (0.34) | 1 (0.91) | 6 (0.50) | None |
| One-stage IPD-AgD NMA – CD4 + HIV RNA | Random | 10 (0.35) | 1 (0.90) | 6 (0.49) | DTG, RAL, EVG/c |
| One-stage IPD-AgD NMA – CD4 + Male | Random | 10 (0.35) | 1 (0.90) | 6 (0.48) | DTG, RAL, EVG/c |
| One-stage IPD-AgD NMA – HIV RNA + Male | Fixed | 10 (0.33) | 1 (0.92) | 6 (0.48) | None |
| One-stage IPD-AgD NMA – CD4 + HIV RNA + Male | Fixed | 10 (0.32) | 1 (0.92) | 6 (0.47) | DTG, RAL, EVG/c |
| Two-stage empirical-priors – CD4 | Random | 10 (0.35) | 1 (0.90) | 6 (0.49) | None |
| Two-stage empirical-priors – HIV RNA | Fixed | 10 (0.28) | 1 (0.90) | 6 (0.48) | DTG, EVG/c, DOR |
| Two-stage empirical-priors – Male | Fixed | 10 (0.33) | 1 (0.91) | 6 (0.48) | None |
| Two-stage empirical-priors – CD4 + HIV RNA | Random | 10 (0.35) | 1 (0.89) | 7 (0.50) | DTG, EVG/c, RPV |
| Two-stage empirical-priors – CD4 + Male | Random | 10 (0.33) | 1 (0.87) | 8 (0.51) | DTG, EVG/c, DRV/r |
| Two-stage empirical-priors – HIV RNA + Male | Fixed | 10 (0.28) | 1 (0.90) | 6 (0.47) | DTG, EVG/c, DOR |
| Two-stage empirical-priors – CD4 + HIV RNA + Male | Fixed | 10 (0.31) | 1 (0.89) | 8 (0.48) | DTG, EVG/c, DRV/r |
| HMR IPD-AgD NMA – CD4 | Random | 10 (0.36) | 1 (0.90) | 6 (0.47) | None |
| HMR IPD-AgD NMA – HIV RNA | Random | 10 (0.34) | 1 (0.91) | 6 (0.47) | None |
| HMR IPD-AgD NMA – Male | Fixed | 11 (0.08) | 1 (0.91) | 9 (0.39) | None |

AgD: Aggregate data; IPD: Individual patient data; NMA: Network meta-analysis; EFV: Efavirenz; DTG: Dolutegravir; EFV_400_: Low-dose efavirenz; RAL: Raltegravir; EVG/c: Cobicistat-boosted elvitegravir; RPV: Rilpivirine; DOR: Doravirine

Table 13: Comparison of comparative treatment estimates for discontinuations

| Analyses | Model | DTG vs. EFV  OR (95% CrI) | EFV_400_ vs. EFV  OR (95% CrI) | DTG vs. EFV_400_  OR (95% CrI) | | Mean change in log-odds | | Maximum change in log-odds | Mean  change in proportion | Maximum change in proportion |
| --- | --- | --- | --- | --- | --- | --- | --- | --- | --- | --- |
| AgD NMA – Unadjusted | Random | 0.52 (0.39, 0.70) | 0.91 (0.46, 1.84) | | 0.57 (0.27, 1.23) | | -- | -- | -- | -- |
| AgD NMA meta-regression – CD4 | Random | 0.55 (0.40, 0.74) | 0.91 (0.46, 1.83) | | 0.60 (0.28, 1.29) | | 0.029 | 0.055 | 0.004 | 0.007 |
| AgD NMA meta-regression – HIV RNA | Fixed | 0.51 (0.40, 0.64) | 0.86 (0.47, 1.60) | | 0.59 (0.31, 1.14) | | 0.047 | 0.101 | 0.005 | 0.016 |
| AgD NMA meta-regression – Male | Random | 0.54 (0.40, 0.71) | 1.06 (0.53, 2.13) | | 0.50 (0.24, 1.04) | | 0.061 | 0.16 | 0.009 | 0.033 |
| Two-stage AgD NMA – CD4 | Random | 0.53 (0.36, 0.77) | 0.92 (0.42, 1.97) | | 0.57 (0.24, 1.37) | | 0.045 | 0.176 | 0.011 | 0.026 |
| Two-stage AgD NMA – HIV RNA | Random | 0.54 (0.37, 0.78) | 0.91 (0.43, 1.96) | | 0.59 (0.25, 1.38) | | 0.048 | 0.168 | 0.011 | 0.025 |
| Two-stage AgD NMA – Male | Random | 0.57 (0.38, 0.85) | 0.91 (0.40, 2.10) | | 0.63 (0.24, 1.59) | | 0.064 | 0.244 | 0.014 | 0.039 |
| Two-stage AgD NMA – CD4 + HIV RNA | Random | 0.59 (0.40, 0.88) | 0.92 (0.41, 2.04) | | 0.65 (0.27, 1.61) | | 0.059 | 0.215 | 0.011 | 0.032 |
| Two-stage AgD NMA – CD4 + Male | Random | 0.57 (0.39, 0.83) | 0.92 (0.42, 2.00) | | 0.62 (0.26, 1.49) | | 0.059 | 0.182 | 0.012 | 0.027 |
| Two-stage AgD NMA – HIV RNA + Male | Random | 0.56 (0.39, 0.81) | 0.92 (0.43, 1.96) | | 0.61 (0.26, 1.43) | | 0.046 | 0.186 | 0.012 | 0.028 |
| Two-stage AgD NMA – CD4 + HIV RNA + Male | Random | 0.59 (0.40, 0.85) | 0.92 (0.42, 1.99) | | 0.64 (0.27, 1.52) | | 0.05 | 0.189 | 0.011 | 0.028 |
| One-stage IPD-AgD NMA – unadjusted | Random | 0.62 (0.46, 0.83) | 0.91 (0.45, 1.81) | | 0.69 (0.32, 1.44) | | 0.048 | 0.175 | 0.026 | 0.044 |
| One-stage IPD-AgD NMA – CD4 | Random | 0.63 (0.46, 0.86) | 0.92 (0.45, 1.90) | | 0.69 (0.31, 1.51) | | 0.057 | 0.193 | 0.012 | 0.023 |
| One-stage IPD-AgD NMA – HIV RNA | Fixed | 0.63 (0.50, 0.79) | 0.91 (0.49, 1.69) | | 0.69 (0.36, 1.33) | | 0.049 | 0.186 | 0.007 | 0.016 |
| One-stage IPD-AgD NMA – Male | Random | 0.62 (0.46, 0.83) | 0.90 (0.44, 1.82) | | 0.68 (0.32, 1.47) | | 0.048 | 0.169 | 0.009 | 0.014 |
| One-stage IPD-AgD NMA – CD4 + HIV RNA | Random | 0.64 (0.46, 0.87) | 0.92 (0.45, 1.93) | | 0.69 (0.31, 1.52) | | 0.061 | 0.198 | 0.006 | 0.02 |
| One-stage IPD-AgD NMA – CD4 + Male | Random | 0.63 (0.46, 0.87) | 0.93 (0.45, 1.93) | | 0.68 (0.31, 1.52) | | 0.059 | 0.194 | 0.009 | 0.015 |
| One-stage IPD-AgD NMA – HIV RNA + Male | Fixed | 0.63 (0.50, 0.80) | 0.91 (0.49, 1.70) | | 0.69 (0.36, 1.34) | | 0.05 | 0.189 | 0.014 | 0.03 |
| One-stage IPD-AgD NMA – CD4+HIV RNA + Male | Fixed | 0.63 (0.50, 0.80) | 0.92 (0.50, 1.70) | | 0.69 (0.36, 1.32) | | 0.052 | 0.191 | 0.011 | 0.028 |
| Two-stage empirical-priors – CD4 | Random | 0.65 (0.46, 0.91) | 0.93 (0.44, 1.96) | | 0.70 (0.30, 1.56) | | 0.103 | 0.265 | 0.014 | 0.029 |
| Two-stage empirical-priors – HIV RNA | Fixed | 0.64 (0.50, 0.80) | 0.89 (0.48, 1.64) | | 0.71 (0.37, 1.38) | | 0.054 | 0.198 | 0.013 | 0.036 |
| Two-stage empirical-priors – Male | Fixed | 0.64 (0.50, 0.81) | 0.92 (0.50, 1.71) | | 0.69 (0.36, 1.33) | | 0.057 | 0.199 | 0.015 | 0.032 |
| Two-stage empirical-priors – CD4 + HIV RNA | Random | 0.66 (0.47, 0.92) | 0.93 (0.46, 1.93) | | 0.71 (0.31, 1.54) | | 0.105 | 0.252 | 0.016 | 0.033 |
| Two-stage empirical-priors – CD4 + Male | Random | 0.66 (0.48, 0.92) | 0.90 (0.44, 1.85) | | 0.74 (0.33, 1.62) | | 0.117 | 0.242 | 0.016 | 0.038 |
| Two-stage empirical-priors – HIV RNA + Male | Fixed | 0.63 (0.49, 0.81) | 0.90 (0.48, 1.66) | | 0.70 (0.36, 1.38) | | 0.057 | 0.188 | 0.017 | 0.041 |
| Two-stage empirical-priors – CD4+HIV RNA+Male | Fixed | 0.67 (0.51, 0.88) | 0.92 (0.49, 1.69) | | 0.73 (0.37, 1.45) | | 0.123 | 0.252 | 0.017 | 0.052 |
| HMR IPD-AgD NMA – CD4 | Random | 0.62 (0.40, 0.94) | 0.92 (0.43, 1.97) | | 0.67 (0.32, 1.44) | | 0.043 | 0.171 | 0.026 | 0.039 |
| HMR IPD-AgD NMA – HIV RNA | Random | 0.45 (0.17, 3.80) | 0.73 (0.25, 6.32) | | 0.64 (0.29, 1.37) | | 0.239 | 0.294 | 0.088 | 0.147 |
| HMR IPD-AgD NMA – Male | Fixed | 0.36 (0.22, 0.57) | 0.61 (0.30, 1.23) | | 0.59 (0.30, 1.15) | | 0.491 | 0.58 | 0.028 | 0.046 |

AgD: Aggregate data; IPD: Individual patient data; NMA: Network meta-analysis; EFV: Efavirenz; DTG: Dolutegravir; EFV_400_: Low-dose efavirenz; OR: Odds ratio; CrI: Credible interval.

**Table 14: Coefficient estimates for discontinuations**

| Analyses | Model | ß_1,1_  Median  (95% CrI) | ß_1,2_  Median  (95% CrI) | ß_1,3_  Median  (95% CrI) | ß_0,1_  Median  (95% CrI) | ß_0,2_  Median  (95% CrI) | ß_0,3_  Median  (95% CrI) |
| --- | --- | --- | --- | --- | --- | --- | --- |
| AgD NMA meta-regression – CD4 | Fixed | 0.059  (-0.052, 0.17) | -- | -- | -- | -- | -- |
| AgD NMA meta-regression – HIV RNA | Fixed | -0.578  (-1.192, 0.032) | -- | -- | -- | -- | -- |
| AgD NMA meta-regression – Male | Fixed | 0.981  (-0.115, 2.044) | -- | -- | -- | -- | -- |
| One-stage IPD-AgD NMA – CD4 | Fixed | -0.063  (-0.193, 0.055) | -- | -- | 0.288  (-1.891, 1.52) | -- | -- |
| One-stage IPD-AgD NMA – HIV RNA | Fixed | -0.02  (-0.326, 0.296) | -- | -- | 0.886  (-3.025, 4.444) | -- | -- |
| One-stage IPD-AgD NMA – Male | Fixed | -0.089  (-0.665, 0.506) | -- | -- | 1.356  (-6.661, 10.092) | -- | -- |
| One-stage IPD-AgD NMA – CD4 + HIV RNA | Fixed | -0.017  (-0.331, 0.295) | 0.011  (-0.535, 0.587) | -- | -0.321  (-6.972, 16.569) | -2.568  (-32.086, 17.175) | -- |
| One-stage IPD-AgD NMA – CD4 + Male | Fixed | -0.064  (-0.194, 0.055) | -0.074  (-0.653, 0.536) | -- | 0.213  (-1.757, 3.197) | 2.442  (-5.847, 10.903) |  |
| One-stage IPD-AgD NMA – HIV RNA + Male | Fixed | -0.065  (-0.201, 0.061) | 0.007  (-0.345, 0.36) | -- | -0.192  (-2.914, 2.967) | 0.311  (-3.718, 5.971) | -- |
| One-stage IPD-AgD NMA – CD4 + HIV RNA + Male | Fixed | -0.036  (-0.152, 0.078) | -0.058  (-0.398, 0.285) | 0.052  (-0.504, 0.651) | -0.254  (-4.894, 2.725) | -10.091  (-22.169, -2.213) | 23.074  (10.576, 36.243) |

*Covariates 1-3 represents the covariates as ordered in the descriptor. E.g., in the last row 1 represents CD4, 2 represents HIV RNA and 3 represents male.*

Table 15: Comparison of model selection and fit for discontinuations due to adverse events

| Analyses | Model | DIC | pD | Deviance | Between study | prop3 | prop4 |
| --- | --- | --- | --- | --- | --- | --- | --- |
| AgD NMA – Unadjusted | Fixed | 205.79 | 66.95 | 138.84 | 0.213 (0.019, 0.503) | 9/116 | 3/116 |
| AgD NMA meta-regression – CD4 | Fixed | 205.27 | 67.89 | 137.38 | 0.21 (0.007, 0.498) | 8/116 | 4/116 |
| AgD NMA meta-regression – HIV RNA | Fixed | 207.20 | 67.89 | 139.31 | 0.214 (0.013, 0.498) | 7/116 | 3/116 |
| AgD NMA meta-regression – Male | Fixed | 205.34 | 67.98 | 137.36 | 0.209 (0.016, 0.497) | 8/116 | 3/116 |
| Two-stage AgD NMA – CD4 | Fixed | 206.12 | 66.98 | 139.14 | 0.227 (0.015, 0.509) | 9/116 | 3/116 |
| Two-stage AgD NMA – HIV RNA | Fixed | 205.79 | 66.85 | 138.94 | 0.219 (0.013, 0.501) | 10/116 | 3/116 |
| Two-stage AgD NMA – Male | Fixed | 205.88 | 66.87 | 139.01 | 0.225 (0.013, 0.505) | 9/116 | 3/116 |
| Two-stage AgD NMA – CD4 + HIV RNA | Fixed | 205.98 | 66.89 | 139.09 | 0.228 (0.017, 0.506) | 10/116 | 3/116 |
| Two-stage AgD NMA – CD4 + Male | Fixed | 205.89 | 67.00 | 138.89 | 0.223 (0.021, 0.515) | 9/116 | 3/116 |
| Two-stage AgD NMA – HIV RNA + Male | Fixed | 205.65 | **66.82** | 138.83 | 0.22 (0.017, 0.502) | 9/116 | 3/116 |
| Two-stage AgD NMA – CD4 + HIV RNA + Male | Fixed | 205.85 | 66.95 | 138.90 | 0.217 (0.022, 0.502) | 9/116 | 3/116 |
| One-stage IPD-AgD NMA – unadjusted | Fixed | 204.22 | 66.95 | 137.27 | 0.171 (0.004, 0.474) | 9/116 | 3/116 |
| One-stage IPD-AgD NMA – CD4 | Fixed | 204.35 | 67.93 | 136.42 | 0.199 (0.01, 0.476) | 10/116 | 3/116 |
| One-stage IPD-AgD NMA – HIV RNA | Fixed | 205.02 | 67.87 | 137.15 | 0.189 (0.014, 0.477) | 8/116 | 3/116 |
| One-stage IPD-AgD NMA – Male | Fixed | 203.41 | 67.88 | 135.53 | 0.177 (0.007, 0.469) | 9/116 | 3/116 |
| One-stage IPD-AgD NMA – CD4 + HIV RNA | Fixed | 204.25 | 68.86 | 135.39 | 0.194 (0.017, 0.47) | 9/116 | 3/116 |
| One-stage IPD-AgD NMA – CD4 + Male | Fixed | 204.33 | 68.95 | 135.38 | 0.177 (0.007, 0.444) | 6/116 | 4/116 |
| One-stage IPD-AgD NMA – HIV RNA + Male | Fixed | 204.30 | 68.74 | 135.56 | 0.178 (0.01, 0.452) | 6/116 | 3/116 |
| One-stage IPD-AgD NMA – CD4 + HIV RNA + Male | Fixed | 204.52 | 69.98 | 134.54 | 0.156 (0.006, 0.445) | 7/116 | 4/116 |
| Two-stage empirical-priors – CD4 | Fixed | **202.79** | 69.53 | 133.26 | 0.183 (0.015, 0.459) | 9/116 | 4/116 |
| Two-stage empirical-priors – HIV RNA | Fixed | 204.90 | 68.79 | 136.11 | 0.183 (0.005, 0.471) | 8/116 | 3/116 |
| Two-stage empirical-priors – Male | Fixed | 204.43 | 70.16 | 134.27 | 0.172 (0.007, 0.455) | 9/116 | 3/116 |
| Two-stage empirical-priors – CD4 + HIV RNA | Fixed | 202.68 | 72.54 | 130.14 | 0.142 (0.006, 0.43) | 7/116 | 3/116 |
| Two-stage empirical-priors – CD4 + Male | Fixed | 203.37 | 71.32 | 132.05 | 0.173 (0.008, 0.463) | 6/116 | 3/116 |
| Two-stage empirical-priors –HIV RNA+ Male | Fixed | 205.83 | 71.82 | 134.01 | 0.147 (0.01, 0.446) | 6/116 | 3/116 |
| Two-stage empirical-priors – CD4 + HIV RNA + Male | Fixed | 203.76 | 75.28 | **128.48** | 0.130 (0.018, 0.413) | 6/116 | 3/116 |
| HMR IPD-AgD NMA – CD4 | Fixed | 205.23 | 68.36 | 136.87 | 0.197 (0.009, 0.477) | 9/116 | 3/116 |
| HMR IPD-AgD NMA – HIV RNA | Fixed | 204.72 | 67.52 | 137.20 | 0.214 (0.014, 0.516) | 8/116 | 3/116 |
| HMR IPD-AgD NMA – Male | Fixed | 203.34 | 69.42 | 133.92 | 0.172 (0.014, 0.45) | 9/116 | 3/116 |

AgD: Aggregate data; IPD: Individual patient data; NMA: Network meta-analysis; DIC: Deviance information criterion; pD: Effective number of parameters; prop3: Proportion of observations above deviance^2^ + leverage = 3; prop4: Proportion of observations above deviance^2^ + leverage = 4. Between-study heterogeneity obtained through the random-effects model, not the fixed-effect model if it was selected.

Table 16: Comparison of surface under the cumulative ranking (SUCRA) for discontinuations due to adverse events

| Analyses | Model | SUCRA for EFV  Rank (SUCRA) | SUCRA for DTG  Rank (SUCRA) | SUCRA for EFV_400_  Rank (SUCRA) | Change in top 3 ranked |
| --- | --- | --- | --- | --- | --- |
| AgD NMA – Unadjusted | Fixed | 10 (0.20) | 1 (0.90) | 5 (0.68) | DTG, RAL, RPV |
| AgD NMA meta-regression – CD4 | Fixed | 10 (0.20) | 1 (0.86) | 3 (0.72) | DTG, RPV, EFV_400_ |
| AgD NMA meta-regression – HIV RNA | Fixed | 10 (0.22) | 1 (0.86) | 4 (0.70) | DTG, RPV, DOR |
| AgD NMA meta-regression – Male | Fixed | 10 (0.23) | 1 (0.89) | 7 (0.53) | DTG, RPV, DOR |
| Two-stage AgD NMA – CD4 | Fixed | 10 (0.20) | 1 (0.90) | 5 (0.67) | None |
| Two-stage AgD NMA – HIV RNA | Fixed | 10 (0.20) | 1 (0.90) | 5 (0.68) | None |
| Two-stage AgD NMA – Male | Fixed | 10 (0.20) | 1 (0.90) | 5 (0.67) | None |
| Two-stage AgD NMA – CD4 + HIV RNA | Fixed | 10 (0.20) | 1 (0.90) | 5 (0.67) | None |
| Two-stage AgD NMA – CD4 + Male | Fixed | 10 (0.20) | 1 (0.90) | 5 (0.68) | None |
| Two-stage AgD NMA – HIV RNA + Male | Fixed | 10 (0.20) | 1 (0.90) | 5 (0.68) | None |
| Two-stage AgD NMA – CD4 + HIV RNA + Male | Fixed | 10 (0.20) | 1 (0.90) | 5 (0.68) | None |
| One-stage IPD-AgD NMA – unadjusted | Fixed | 10 (0.21) | 1 (0.89) | 4 (0.70) | DTG, RPV, DOR |
| One-stage IPD-AgD NMA – CD4 | Fixed | 10 (0.24) | 1 (0.85) | 4 (0.74) | None |
| One-stage IPD-AgD NMA – HIV RNA | Fixed | 10 (0.21) | 1 (0.86) | 5 (0.71) | DTG, RAL, DOR |
| One-stage IPD-AgD NMA – Male | Fixed | 10 (0.26) | 1 (0.85) | 2 (0.78) | DTG, EFV_400_, RAL |
| One-stage IPD-AgD NMA – CD4 + HIV RNA | Fixed | 9 (0.27) | 1 (0.85) | 2 (0.79) | DTG, EFV_400_, RAL |
| One-stage IPD-AgD NMA – CD4 + Male | Fixed | 10 (0.22) | 1 (0.87) | 3 (0.75) | DTG, RAL, EFV_400_ |
| One-stage IPD-AgD NMA – HIV RNA + Male | Fixed | 10 (0.24) | 1 (0.86) | 3 (0.77) | DTG, RAL, EFV_400_ |
| One-stage IPD-AgD NMA – CD4 + HIV RNA + Male | Fixed | 10 (0.25) | 1 (0.86) | 3 (0.79) | DTG, RAL, EFV_400_ |
| Two-stage empirical-priors – CD4 | Fixed | 10 (0.25) | 1 (0.87) | 4 (0.75) | None |
| Two-stage empirical-priors – HIV RNA | Fixed | 10 (0.23) | 1 (0.85) | 5 (0.71) | DTG, RAL, DOR |
| Two-stage empirical-priors – Male | Fixed | 10 (0.23) | 2 (0.80) | 4 (0.73) | RAL, DTG, RPV |
| Two-stage empirical-priors – CD4 + HIV RNA | Fixed | 10 (0.25) | 2 (0.81) | 3 (0.76) | RAL, DTG, RPV |
| Two-stage empirical-priors – CD4 + Male | Fixed | 10 (0.24) | 1 (0.87) | 3 (0.76) | DTG, RAL, EFV_400_ |
| Two-stage empirical-priors – HIV RNA + Male | Fixed | 10 (0.23) | 2 (0.81) | 5 (0.72) | RAL, DTG, DOR |
| Two-stage empirical-priors – CD4 + HIV RNA + Male | Fixed | 10 (0.24) | 2 (0.82) | 3 (0.75) | RAL, DTG, EFV_400_ |
| HMR IPD-AgD NMA – CD4 | Fixed | 9 (0.32) | 1 (0.85) | 4 (0.73) | None |
| HMR IPD-AgD NMA – HIV RNA | Fixed | 8 (0.40) | 1 (0.83) | 5 (0.70) | None |
| HMR IPD-AgD NMA – Male | Fixed | 6 (0.60) | 2 (0.80) | 1 (0.81) | EFV_400_, DTG, RPV |

AgD: Aggregate data; IPD: Individual patient data; NMA: Network meta-analysis; EFV: Efavirenz; DTG: Dolutegravir; EFV_400_: Low-dose efavirenz; RAL: Raltegravir; EVG/c: Cobicistat-boosted elvitegravir; RPV: Rilpivirine; DOR: Doravirine.

Table 17: Comparison of comparative treatment estimates for discontinuations due to adverse events

| Analyses | Model | DTG vs. EFV  OR (95% CrI) | EFV_400_ vs. EFV  OR (95% CrI) | DTG vs. EFV_400_  OR (95% CrI) | | Mean change in log-odds | | Maximum change in log-odds | Mean change in proportion | Maximum change in proportion |
| --- | --- | --- | --- | --- | --- | --- | --- | --- | --- | --- |
| AgD NMA – Unadjusted | Fixed | 0.28 (0.17, 0.44) | 0.42 (0.22, 0.77) | | 0.67 (0.30, 1.45) | | -- | -- | -- | -- |
| AgD NMA meta-regression – CD4 | Fixed | 0.30 (0.19, 0.48) | 0.40 (0.21, 0.74) | | 0.76 (0.35, 1.65) | | 0.084 | 0.215 | 0.002 | 0.007 |
| AgD NMA meta-regression – HIV RNA | Fixed | 0.30 (0.19, 0.47) | 0.42 (0.22, 0.77) | | 0.72 (0.33, 1.55) | | 0.083 | 0.308 | 0.003 | 0.007 |
| AgD NMA meta-regression – Male | Fixed | 0.27 (0.17, 0.44) | 0.58 (0.27, 1.20) | | 0.47 (0.19, 1.24) | | 0.107 | 0.321 | 0.004 | 0.011 |
| Two-stage AgD NMA – CD4 | Fixed | 0.28 (0.17, 0.44) | 0.42 (0.22, 0.77) | | 0.66 (0.31, 1.47) | | 0.003 | 0.009 | 0 | 0 |
| Two-stage AgD NMA – HIV RNA | Fixed | 0.28 (0.17, 0.44) | 0.42 (0.22, 0.77) | | 0.67 (0.31, 1.47) | | 0.004 | 0.008 | 0 | 0 |
| Two-stage AgD NMA – Male | Fixed | 0.28 (0.17, 0.44) | 0.42 (0.22, 0.77) | | 0.66 (0.30, 1.47) | | 0.004 | 0.008 | 0 | 0.001 |
| Two-stage AgD NMA – CD4 + HIV RNA | Fixed | 0.28 (0.17, 0.44) | 0.42 (0.22, 0.77) | | 0.66 (0.30, 1.47) | | 0.005 | 0.012 | 0 | 0.001 |
| Two-stage AgD NMA – CD4 + Male | Fixed | 0.28 (0.17, 0.44) | 0.42 (0.22, 0.77) | | 0.66 (0.30, 1.47) | | 0.003 | 0.01 | 0 | 0 |
| Two-stage AgD NMA – HIV RNA + Male | Fixed | 0.28 (0.17, 0.45) | 0.42 (0.22, 0.76) | | 0.66 (0.31, 1.50) | | 0.004 | 0.009 | 0 | 0.001 |
| Two-stage AgD NMA – CD4 + HIV RNA + Male | Fixed | 0.28 (0.17, 0.45) | 0.42 (0.22, 0.76) | | 0.67 (0.31, 1.47) | | 0.004 | 0.008 | 0 | 0 |
| One-stage IPD-AgD NMA – unadjusted | Fixed | 0.35 (0.22, 0.52) | 0.42 (0.22, 0.77) | | 0.82 (0.39, 1.79) | | 0.094 | 0.24 | 0.011 | 0.027 |
| One-stage IPD-AgD NMA – CD4 | Fixed | 0.37 (0.23, 0.57) | 0.43 (0.22, 0.79) | | 0.85 (0.41, 1.85) | | 0.144 | 0.305 | 0.006 | 0.017 |
| One-stage IPD-AgD NMA – HIV RNA | Fixed | 0.32 (0.20, 0.49) | 0.40 (0.21, 0.74) | | 0.80 (0.37, 1.72) | | 0.056 | 0.165 | 0.007 | 0.019 |
| One-stage IPD-AgD NMA – Male | Fixed | 0.36 (0.23, 0.55) | 0.39 (0.20, 0.72) | | 0.92 (0.43, 2.00) | | 0.159 | 0.298 | 0.002 | 0.005 |
| One-stage IPD-AgD NMA – CD4 + HIV RNA | Fixed | 0.38 (0.24, 0.58) | 0.40 (0.21, 0.74) | | 0.94 (0.44, 2.04) | | 0.193 | 0.338 | 0.002 | 0.005 |
| One-stage IPD-AgD NMA – CD4 + Male | Fixed | 0.33 (0.21, 0.53) | 0.40 (0.21, 0.74) | | 0.83 (0.39, 1.79) | | 0.086 | 0.206 | 0.005 | 0.013 |
| One-stage IPD-AgD NMA – HIV RNA + Male | Fixed | 0.33 (0.21, 0.52) | 0.37 (0.19, 0.70) | | 0.89 (0.41, 1.93) | | 0.109 | 0.213 | 0.003 | 0.008 |
| One-stage IPD-AgD NMA – CD4+ HIV RNA+ Male | Fixed | 0.34 (0.21, 0.54) | 0.38 (0.20, 0.70) | | 0.91 (0.42, 1.98) | | 0.141 | 0.238 | 0.003 | 0.007 |
| Two-stage empirical-priors – CD4 | Fixed | 0.37 (0.23, 0.58) | 0.44 (0.23, 0.81) | | 0.84 (0.39, 1.85) | | 0.205 | 0.588 | 0.01 | 0.028 |
| Two-stage empirical-priors – HIV RNA | Fixed | 0.32 (0.21, 0.50) | 0.41 (0.22, 0.76) | | 0.79 (0.37, 1.74) | | 0.076 | 0.232 | 0.009 | 0.025 |
| Two-stage empirical-priors – Male | Fixed | 0.37 (0.23, 0.58) | 0.41 (0.22, 0.76) | | 0.90 (0.42, 1.97) | | 0.097 | 0.288 | 0.01 | 0.028 |
| Two-stage empirical-priors – CD4 + HIV RNA | Fixed | 0.40 (0.25, 0.66) | 0.43 (0.23, 0.80) | | 0.93 (0.42, 2.11) | | 0.199 | 0.648 | 0.011 | 0.029 |
| Two-stage empirical-priors – CD4 + Male | Fixed | 0.35 (0.22, 0.57) | 0.43 (0.22, 0.79) | | 0.83 (0.38, 1.87) | | 0.167 | 0.582 | 0.01 | 0.028 |
| Two-stage empirical-priors – HIV RNA + Male | Fixed | 0.34 (0.21, 0.56) | 0.41 (0.21, 0.75) | | 0.85 (0.38, 1.90) | | 0.105 | 0.264 | 0.01 | 0.029 |
| Two-stage empirical-priors – CD4+HIV RNA+Male | Fixed | 0.38 (0.24, 0.66) | 0.43 (0.22, 0.77) | | 0.90 (0.42, 2.15) | | 0.167 | 0.544 | 0.01 | 0.03 |
| HMR IPD-AgD NMA – CD4 | Fixed | 0.42 (0.20, 0.79) | 0.50 (0.22, 1.05) | | 0.84 (0.40, 1.80) | | 0.288 | 0.423 | 0.004 | 0.01 |
| HMR IPD-AgD NMA – HIV RNA | Fixed | 0.48 (0.11, 1.51) | 0.57 (0.15, 1.94) | | 0.82 (0.39, 1.78) | | 0.395 | 0.542 | 0.011 | 0.029 |
| HMR IPD-AgD NMA – Male | Fixed | 0.76 (0.28, 1.75) | 0.71 (0.30, 1.59) | | 1.05 (0.47, 2.42) | | 0.866 | 1.035 | 0.04 | 0.092 |

AgD: Aggregate data; IPD: Individual patient data; NMA: Network meta-analysis; EFV: Efavirenz; DTG: Dolutegravir; EFV_400_: Low-dose efavirenz; OR: Odds ratio; CrI: Credible interval.

**Table 18: Coefficient estimates for discontinuations due to adverse events**

| Analyses | Model | ß_1,1_  Median  (95% CrI) | ß_1,2_  Median  (95% CrI) | ß_1,3_  Median  (95% CrI) | ß_0,1_  Median  (95% CrI) | ß_0,2_  Median  (95% CrI) | ß_0,3_  Median  (95% CrI) |
| --- | --- | --- | --- | --- | --- | --- | --- |
| AgD NMA meta-regression – CD4 | Fixed | -0.187  (-0.447, 0.072) | -- | -- | -- | -- | -- |
| AgD NMA meta-regression – HIV RNA | Fixed | -0.288  (-1.468, 0.884) | -- | -- | -- | -- | -- |
| AgD NMA meta-regression – Male | Fixed | 2.079  (-0.668, 4.808) | -- | -- | -- | -- | -- |
| One-stage IPD-AgD NMA – CD4 | Fixed | -0.345  (-0.955, 0.266) | -- | -- | 1.156  (-2.548, 4.578) | -- | -- |
| One-stage IPD-AgD NMA – HIV RNA | Fixed | -0.942  (-1.863, 0.095) | -- | -- | 3.233  (-3.894, 11.112) | -- | -- |
| One-stage IPD-AgD NMA – Male | Fixed | -0.111  (-0.317, 0.09) | -- | -- | -0.432  (-1.521, 1.293) | -- | -- |
| One-stage IPD-AgD NMA – CD4 + HIV RNA | Fixed | -0.323  (-0.932, 0.278) | -0.914  (-1.827, 0.119) | -- | 2.126  (-9.166, 9.198) | -1.718  (-17.53, 20.984) | -- |
| One-stage IPD-AgD NMA – CD4 + Male | Fixed | -0.088  (-0.294, 0.108) | -0.885  (-1.831, 0.178) | -- | 0.944  (-1.301, 3.468) | 6.132  (-3.154, 13.337) | -- |
| One-stage IPD-AgD NMA – HIV RNA + Male | Fixed | -0.168  (-0.391, 0.046) | -0.511  (-1.157, 0.131) | -- | 0.709  (-1.755, 4.184) | 2.851  (-2.284, 8.213) | -- |
| One-stage IPD-AgD NMA – CD4 + HIV RNA + Male | Fixed | -0.143  (-0.366, 0.069) | -0.468  (-1.107, 0.168) | -0.815  (-1.767, 0.254) | 0.053  (-5.472, 2.353) | -3.287  (-32.082, 7.516) | 10.993  (-4.278, 52.43) |

*Covariates 1-3 represents the covariates as ordered in the descriptor. E.g., in the last row 1 represents CD4, 2 represents HIV RNA and 3 represents male.*

###

### References

1. Dempster A. The direct use of likelihood for significance testing. Statistics and Computing. 1997; 7 (4): 247-52.

2. Spiegelhalter DJ, Best NG, Carlin BP, Van Der Linde A. Bayesian measures of model complexity and fit. J R Stat Soc B. 2002; 64 (4): 583-639.

3. Salanti G, Ades AE, Ioannidis JP. Graphical methods and numerical summaries for presenting results from multiple-treatment meta-analysis: an overview and tutorial. J Clin Epidemiol. 2011; 64 (2): 163-71.

4. Dias S, Sutton AJ, Ades AE, Welton NJ. Technical Support Document 2: A generalized linear modelling framework for pairwise and network meta-analysis of randomized controlled trials. Med Decis Making. 2013; 33 (5): 607-17.
